# Post-transcriptional control of *KRAS*: functional roles of 5′UTR RNA G-quadruplexes, long noncoding RNA, and hnRNPA1

**DOI:** 10.1093/nar/gkaf886

**Published:** 2025-09-18

**Authors:** Ylenia Cortolezzis, Zahraa Othman, Francesca Agostini, Iman Ibrahim, Raffaella Picco, Gilmar F Salgado, Eros Di Giorgio, Luigi E Xodo

**Affiliations:** Department of Medicine, Laboratory of Biochemistry, University of Udine, 33100 Udine, Italy; ARNA Laboratory, Université de Bordeaux, Inserm U1212, CNRS UMR 5320, IECB, 33607 Pessac, France; Department of Medicine, Laboratory of Biochemistry, University of Udine, 33100 Udine, Italy; ARNA Laboratory, Université de Bordeaux, Inserm U1212, CNRS UMR 5320, IECB, 33607 Pessac, France; Department of Medicine, Laboratory of Biochemistry, University of Udine, 33100 Udine, Italy; ARNA Laboratory, Université de Bordeaux, Inserm U1212, CNRS UMR 5320, IECB, 33607 Pessac, France; Department of Medicine, Laboratory of Biochemistry, University of Udine, 33100 Udine, Italy; Department of Medicine, Laboratory of Biochemistry, University of Udine, 33100 Udine, Italy

## Abstract

Previous studies have shown that human *KRAS* expression is regulated at the transcriptional level by G-quadruplex DNA structures within its promoter. Here we show an additional level of regulation involving a post-transcriptional mechanism centred on the 5′-untranslated region (5′UTR) of the messenger RNA (mRNA) characterized by G4 structures (rG4s). Long noncoding RNAs (lncRNAs) and the protein hnRNPA1 are also involved in this mechanism. RIP-seq confirmed the presence of rG4s in the 5′UTR. Deletion of the rG4 region using CRISPR/Cas9 resulted in a significant increase in *KRAS* mRNA levels, indicating the role of the 5′UTR in controlling mRNA levels. RIP shows that hnRNPA1 is recruited to the 5′UTR, where it unfolds the rG4 structures and potentially affects mRNA stability. In addition, lncRNAs transcribed from the LINC01750 locus can hybridize to the rG4 region of 5′UTR and form RNA duplexes leading to RNase III-assisted degradation of the targeted mRNA. Activation of the LINC01750 locus with dCas9-VP64 resulted in downregulation of *KRAS* mRNA, whereas its suppression with dCas9-KRAB led to upregulation of both *KRAS* mRNA and protein. Since lncRNA-mediated regulation of mRNA appears to be a crucial aspect of cellular homeostasis and its disruption contributes to various diseases, understanding these mechanisms may reveal promising new therapeutic targets.

## Graphical Abstract

**Graphical Abstract:**
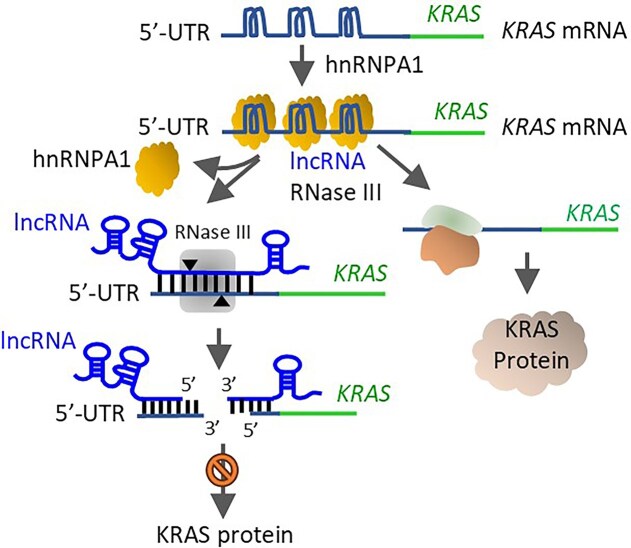

## Introduction

Over the last two decades, considerable efforts have been made to decipher the intricate role of G-quadruplex (G4) structures in cellular processes [1, 2]. G4s are unique nucleic acid secondary structures consisting of guanine-rich sequences [3]. They are stabilized by potassium, the predominant monovalent cation in the intracellular environment, typically at a concentration of 140 mM [4]. Bioinformatic analyses have shown that the distribution of G4 motifs in the genome is not random and that G4 motifs are significantly enriched in the regulatory regions of genes [5–8].

There is increasing evidence that G4 motifs in gene promoters are involved in the regulation of transcription [9–14]. A central question is whether gene regulation is governed by the G4 sequence itself or its folded structure. In other words, the researchers want to find out whether the G4 motifs have evolved as part of a sophisticated epigenetic mechanism that enables precise gene regulation in a cellular context. The presence of G4 DNA in cells has been detected using G4-specific antibodies and small molecule probes [15–17].

Several studies suggest that G4 can directly influence chromatin structure and dynamics [9, 18, 19]. For example, the formation of G4 can hinder the assembly or disassembly of nucleosomes and thus impair the accessibility and compaction of chromatin [20]. G4 structures can also serve as nucleation sites for the formation of higher order chromatin structures [21]. Several studies suggest that G4 structures may differentially affect transcription depending on their location and orientation relative to the transcription start site (TSS) [22]. However, recent mapping of G4 structures in chromatin showed a positive correlation between G4 structures in promoters and active transcription [10]. The idea that G4 structures act as a hub for the recruitment of transcription factors (TFs) to the promoter of the target gene is gaining support among scientists. In addition, G4s can influence the recruitment and activity of chromatin-modifying enzymes such as histone acetyltransferases, methyltransferases and chromatin remodellers, leading to changes in histone modifications and chromatin compaction [23].

Among the G4s that have been the subject of several biophysical and biological studies is the G4 formed in the promoter of the human and murine Kirsten ras (*KRAS*) oncogene [20, 24–26]. Transcriptional regulation of the human *KRAS* gene is controlled by a complex mechanism involving G-rich elements located upstream of the TSS, in particular 32R (−148/−116) and G4-mid (−207/−175) [27]. Extensive research has focused on the 32R G4 motif, which forms a stable G4 structure [24, 27] and serves as a recognition site for nuclear proteins such as PARP-1, MAZ, Ku70, and hnRNPA1, that have been identified by biotin–streptavidin pull-down assays in conjunction with mass spectrometry [25]. The 32R G4 functions as a platform for the recruitment of TFs to the *KRAS* promoter and promotes the assembly of the transcription preinitiation complex [20, 28–30]. The central role of the 32R-G4 motif in promoter functions was also confirmed by the experiment in which the genomic MYC-G4 was replaced by the 32R-G4 structure, successfully restoring MYC transcription [10].

**Figure 1.**
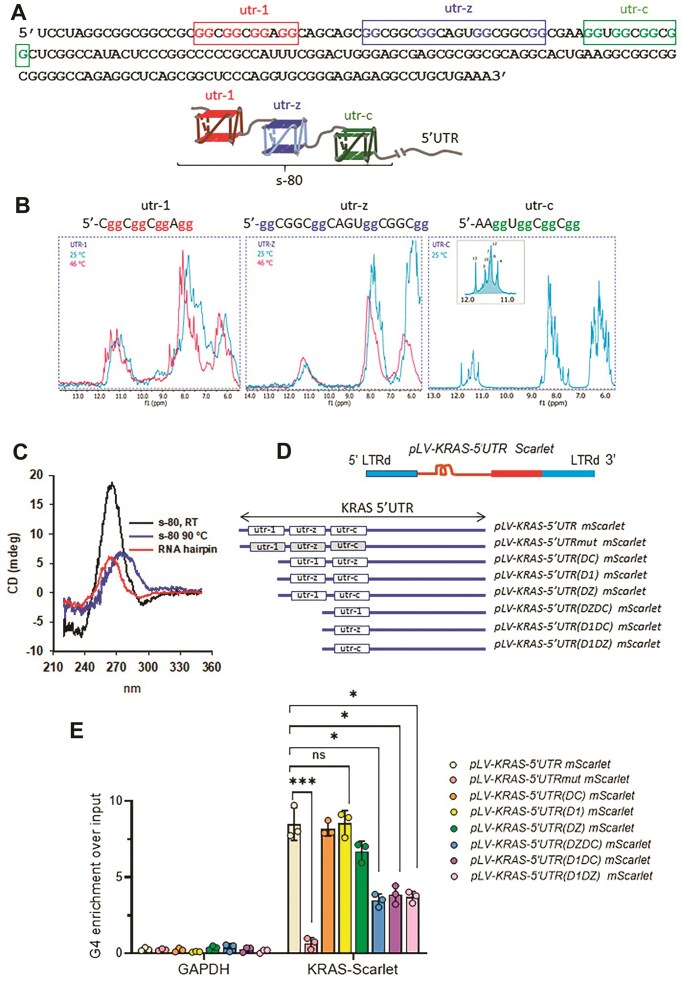
Formation of rG4 structures in the 5′UTR of *KRAS* mRNA. (**A**) Ribonucleotide sequence of the 5′UTR of *KRAS* mRNA (the sequence does not contain the intron and refers to the mature post-splicing form). The cartoon shows the three nonoverlapping rG4 structures formed within the first 80 nt of the 5′UTR of *KRAS* mRNA (s-80). (**B**) Sequences of the rG4 motifs present in the first 80 nt of KRAS 5′UTR and 1H Nuclear magnetic resonance (NMR) imino proton spectra of 200 μM utr-1, utr-z, and utr-c rG4 at 25°C and 46°C in 100 nM KCl, 10 mM KPi buffer. (**C**) Circular dichroism (CD) spectra at 25°C and 85°C of 3 μM s-80 (80-mer oligoribonucleotide) in 50 mM Tris–HCl, pH 7.4, 100 mM KCl. (**D**) Structure of the lentiviral plasmids carrying the KRAS-5′UTR, with the three rG4 motifs, upstream of Scarlet. Construct *pLV-KRAS-5′UTRmut Scarlet* contains the three rG4 motif mutated, whereas the other constructs carry mutational deletions of one or two rG4 motifs, as reported in the figure panel. (**E**) RNA immunoprecipitation (RIP) with G4-specific antibody BG4 in 293T cells stably trasduced with the lentiviral plasmids. The plot shows the rG4 enrichment over input. GAPDH was used as a control. Data are shown as mean ± standard deviation (SD; *n* = 3, independent experiments) (Dunn’s multiple comparison test).

The *KRAS* transcript also contains a G-rich 5′ untranslated region (5′UTR) spanning 193 nucleotides (nt) and with 33 GG blocks, yielding multiple RNA G4 (rG4) motifs (Fig. 1A). Within the first 80 nt of this 5′UTR region, we identified three nonoverlapping sequences, namely utr-1, utr-c, and utr-z, that can form stable rG4 structures [28]. In previous work, we have shown that these rG4 structures can serve as a platform for the binding of small molecules such as anthrathiophenediones [28] or cationic porphyrins [31] that upon illumination generate reactive oxygen species that degrade messenger RNA (mRNA). Despite the recognized role of the 5′UTR region in the regulation of gene expression, this aspect remains unexplored in *KRAS*. Our study aims to fill this gap by investigating the functional significance of the 5′UTR region, in particular the section proximal to the 5′ cap that contains the G4 motifs. This study provides valuable insights into the regulatory role that the 5′UTR plays in modulating the oncogenic potential of *KRAS*.

## Materials and methods

### Cell culture and treatments

Phoenix Ampho (RRID:CVCL_H716), 293T (RRID:CVCL_0045), MIA PaCa-2 (RRID:CVCL_0045), and Panc-1 (RRID:CVCL_0480) were obtained from ATCC (Virginia, USA). Panc-1 hnRNPA1^−/−^ were previously generated and validated by Sanger sequencing [30]. Cells were grown in Dulbecco’s modified Eagle’s medium (Euroclone, Italy) supplemented with 10% fetal bovine serum (FBS), penicillin/streptomycin, and 2 mM L-glutamine (Euroclone, Italy) at 37°C and 5% CO_2_. The following chemicals were used: 0.1, 1.0, 10 μM pyridostatin (PDS; SML2690, Sigma–Aldrich, Germany), 10 μg/ml cyclohexamide (CHX; 66-81-9, Sigma–Aldrich, Germany), 20 nM actinomycin D (ActD; 50-76-0, Sigma–Aldrich, Germany).

### Plasmid construction, transfection, and lentiviral infection

*pLV-mScarlet-Control-5′UTR* (#184637), 3xMEF2-luc (#32967) were purchased from Addgene (MA, USA). pGL3-basic was purchased from Promega (Madison, USA). *pKRASpro* (−377/−1) and *pKRASpro-utr* (−377/+180) were amplified from genomic DNA of BJ cells, restricted with NheI/BglII and cloned into pGL3-basic, which was linearized with NheI/BamHI. 5′UTR *KRAS* reporter was prepared by subcloning *KRAS* 5′UTR (1–180) amplified from *pKRASpro-utr* into pGL3-basic and into *pLV-mScarlet-Control-5′UTR* linearized with BsmbI and AgeI. The core sequences HSALNT0117439 and HSALNT0012722 were chemically synthesized (MWG Eurofins, Germany) and cloned by oligocloning into *pcDNA3* linearized with BamHI and XbaI. Transfection of 293T cells was performed with linear polyethylenimine (L-PEI) (PEI:DNA 4:1), while Panc-1 cells were transfected with Lipofectamine 2000 (LifeTechnologies, Carlsbad, USA) according to the manufacturer’s instructions. To generate the stable Panc-1 *KRAS* 5′-utr reporter cell line, 293T cells seeded in 60 mm plates were transfected with 10 μg *pLV-mScarlet-Control-5′UTR* or *KRAS*-5′UTR, 5 μg psPAX2, 1.8 μg pMD2.G using L-PEI. The virions were harvested 48 and 96 h after transfection and used to infect Panc-1 cells at 0.3 MOI.

### Cas9-assisted deletion of utr-1 and utr-z

*KRAS* 5′UTR-specific single-guide RNAs (sgRNAs; sg1: 5′-TTCCTAGGCGGCGGCCGCGG-3′; sg2: AGTGGCGGCGGCGAAGGTGG) were cloned by oligocloning into pSpCas9(BB)-2A-Puro (PX459) (Addgene, #62988). A total of 3.3 × 10^5^ 293T cells were transfected with 1 μg pSpCas9(BB)-2A-Puro (PX459) containing sgRNA1 and sgRNA2, 100 pmol ssODN (5′-TGCTCGGAG-CTCGATTTTCCTAGGCGGCGGCCGTGGCGGCGGCTCGGCCAGTACTCCCGG-3′) and 5 μl PEI (Merck, Germany). The monoclonal cultures were screened by PCR from genomic DNA (gDNA) (primer FW: GCTCGGAGCTCGATTTTCCTAG, primer RV: CCGGGAGTACTGGCCGAGCCG). A clone with a successful deletion of utr-1 and utr-z was amplified and characterized. No obvious phenotype of reduced fitness was observed during cell cultivation.

### CRISPR/Cas9-mediated transcriptional regulation of LINC01750

To modulate transcriptional activity at the *LINC01750* locus, four sgRNAs were designed using the CRISPOR tool (RRID:SCR_015935):

- sg1: 5′-GGTGGCGGAGTGTACCGACG-3′
- sg2: 5′-GTACGGCGTGAGCGTAAGTT-3′
- sg3: 5′-GCCGCAAAGATTGCGCGGTA-3′
- sg4: 5′-CGAGCCGGTCCCCTTACAAC-3′

These sgRNAs were cloned via oligo annealing into the lentiviral vectors pLenti SAMv2 (Addgene #75112) for transcriptional activation and Lenti-(BB)-EF1α-KRAB-dCas9-P2A-BlastR (Addgene #118154) for transcriptional repression.

Lentiviral particles were produced using standard packaging protocols and used to infect HEK293 and MIA PaCa-2 cells. Post-infection, cells were selected and harvested for downstream expression analysis.

The efficiency of CRISPR-mediated activation or repression of *LINC01750* was evaluated by quantitative reverse transcription PCR (qRT-PCR). Total RNA was extracted, reverse transcribed, and amplified using SYBR Green chemistry (KAPA Biosystems, USA) with the following transcript-specific primers:

- Forward: 5′-GAATCCTGGATCTCGGCCTG-3′
- Reverse: 5′-CACCTCTCCACACCCAGAAA-3′

Expression levels were normalized to housekeeping genes *GAPDH* and *HPRT*, and relative quantification was performed using the ΔΔCt method.

### Generation of *KRAS* 5′UTR reporter cell lines

To investigate the regulatory activity of the *KRAS* 5′UTR, reporter cell lines were generated by cloning the *KRAS* 5′UTR sequence and its deletion mutants upstream of mScarlet reporter gene. The construct was inserted into a lentiviral backbone under the control of a constitutive CMV promoter. Following vector assembly and sequence verification, lentiviral particles were produced and used to transduce target cell lines (293T and Panc-1). Transduced cells were expanded for downstream assays, including reporter activity measurements and RNA stability analyses.

### RNA extraction and quantitative qRT-PCR

Total RNA was extracted by a phenol/chloroform extraction method using Tri reagent (Sigma–Aldrich, Germany). Data were analysed using a comparative threshold cycle (delta delta Ct) using HPRT and/or GAPDH as normalizers or GFP if pEGFPC1 was co-transfected into the cells. For treatment with ActD and cycloheximide (Chx), cells were treated with 20 nM ActD and 10 μg/ml Chx and harvested at the indicated time after treatment. The primers used are listed in Supplementary Table S1.

### 4-Thiouridine metabolic RNA labelling and isolation of newly transcribed RNA

For metabolic labelling of nascent RNA, cells were incubated with 100 μM 4-thiouridine (4sU; Merck, Milan, Italy) for 4 h under standard culture conditions. Total RNA was then extracted using a standard phenol/chloroform protocol followed by ethanol precipitation to ensure high purity and integrity. Biotinylation of 4sU-labelled RNA was performed by incubating 1 μg of total RNA with 2 μl of biotin-HPDP (1 mg/ml in dimethylformamide; Merck, Milan, Italy) in freshly prepared biotinylation buffer [10 mM Tris–HCl (pH 7.4), 1 mM ethylenediaminetetraacetic acid (EDTA)] for 1.5 h at room temperature in the dark. After biotinylation, the unincorporated biotin was removed by chloroform extraction and ethanol precipitation.

To isolate the biotinylated RNA, 100 μg of total RNA was incubated with 16 μl of streptavidin-coated magnetic beads (Promega, USA) for 15 min at room temperature with gentle rotation. The beads were then washed three times with high-salt wash buffer [100 mM Tris–HCl (pH 7.4), 10 mM EDTA, 1 M NaCl, 0.1% Tween-20] to remove nonspecifically bound RNA. Finally, the bound RNA was eluted by incubating the beads with 100 mM dithiothreitol (DTT) and recovered by ethanol precipitation.

### Purification of RNAs interacting with the KRAS 5′UTR

To isolate RNAs interacting with the *KRAS* 5′UTR, 100 μg of total RNA extracted from Panc-1 or 293T cells (the latter transduced with pLenti SAMv2 to overexpress *LINC01750*) were incubated with 10 μg of synthetic biotinylated RNA probes. Two versions of the probe were used:

- Wild-type *KRAS* 5′UTR sequence: 5′-GCAGCGGCGGCGGCAGUGGCGGCGGCGAAGGUGGCGGCGGC-3′
- Mutant rG4-disrupted sequence: 5′-GCAGCGCCAGCCGCAGUCGCGUCCGCGAACGUAGCCGCCGC-3′

RNA mixtures were denatured at 70°C for 5 min and then slowly cooled to room temperature at a rate of 0.5°C per minute to allow proper folding and hybridization.

Following annealing, biotinylated RNA–RNA complexes were captured using 16 μl of streptavidin-coated magnetic beads (Promega, USA) and incubated at room temperature with gentle rotation. Beads were then washed thoroughly with high-salt wash buffer to remove nonspecifically bound RNAs. Finally, bound RNAs were eluted and purified for downstream analysis.

### Luciferase assays

For the experiments in Fig. 3, 293T cells were transfected with 1 μg *pKRASpro, pKRASpro-utr* or *p3xMEF2-luc* and 100ng pRenilla. Cells were then treated as indicated in the figure legends and harvested after 48 h according to the manufacturer’s instructions (Dual-Glo Luciferase Assay System, Promega, USA). Luminescence was quantified using the Modulus II microtiter plate multimode reader (Turner Biosystem, CA, USA) and normalized to Renilla luminescence values accordingly. For the experiments in Fig. 3, 293T cells were transfected with a 1:1 ratio of the described luc reporter and pEGFP C1. As a reference, GFP fluorescence was detected using Synergy H1 (Biotek, US).

### Immunoblotting

Cells were lysed with 2× Laemmli sample buffer [4% sodium dodecyl sulphate (SDS), 20% glycerol, 0.004% bromophenol blue, 0.125 M Tris–Cl (pH 6.8), 10% 2-mercaptoethanol]. The proteins were resolved by sodium dodecyl sulphate–polyacrylamide gel electrophoresis (SDS–PAGE) and immunoblotted on nitrocellulose (Whatman, UK). The films were incubated with the following primary antibodies: anti-ACTIN (Merck, A2066), anti-hnRNPA1 (Merck, 9H10), and anti-KRAS (Merck, 3B10-2F2). Horseradish peroxidase-conjugated secondary antibodies were purchased from Cell Signalling (Danvers, USA) and the blots were developed using Super Signal West Dura (Pierce, USA). AF660 or AF760 secondary antibodies were used for fluorescence detection (Merck, Germany), and images were acquired using the Odyssey M Imaging System (LI-COR Biosciences, USA).

### Recombinant hnRNPA1 and electrophoretic mobility shift assays

Recombinant GST-hnRNPA1 was expressed in *Escherichia coli* BL21 using the plasmid pGEX4T1-hnRNPA1. The bacteria were grown at 37°C until an optical density of 0.8 was reached. Protein expression was induced overnight at 16°C with 0.2 mM Isopropil-β-D-1-tiogalattopiranoside (IPTG) (Merck, Germany). After centrifugation (3000 rcf, 10 min), the bacterial pellet was lysed with GST lysis buffer [phosphate buffered saline (PBS; pH 7.4), 1% Triton X-100; 0.2% SDS, 0.5% NP-40, 0.1% Tween-20, protease inhibitors, 10 ml GST lysis buffer for 250 ml bacterial liquid culture] and sonicated (SonoPlus loop sonicator, five strokes 30 s). The soluble fraction was recovered after centrifugation (10 min at 3500 rpm) and loaded onto Bio-Rad affinity chromatography columns pre-packed with Glutathione Sepharose 4 Fast Flow (GE Healthcare, USA). Elution was performed by competition [50 mM Tris–HCl (pH 8.0), 10 mM L-glutathione reduced (GSH)], followed by dialysis in 50 mM Tris–HCl (pH 7.5), 150 mM NaCl.

For electrophoretic mobility shift assay (EMSA), the Cy5.5 end-labelled oligoribonucleotide utr-c + utr-z (41mer 5′-GCAGCGGCGGCGGCAGUGGCGGCGGCGAAGGUGGCGGCGGC-3′) was allowed to adopt its structure in 50 mM Tris–HCl (pH 7.4) and 100 mM KCl (heated to 95°C for 5 min and annealed at RT). Cy5.5 oligonucleotides (30 nM) were incubated for 15 min at 25°C with increasing amounts of hnRNPA1 in 50 mM Tris–HCl (pH 7.4), 50 mM KCl, 1 mM EDTA, 1 mM Na_3_VO_4_, 5 mM NaF, 1 mM dithiothreitol (DTT), and 8% glycerol. The reaction mixtures were subjected to native gel electrophoresis at 4°C using 8% acrylamide/bisacrylamide gels (19:1 ratio) with 10 mM KCl and 1× Tris-borate-EDTA (TBE) buffer. After the run, the gel was analysed using the Odyssey CLx Imaging System (Li-COR Biosciences, Lincoln, USA).

### Duplex formation and RNase III cleavage

The Cy5.5 end-labelled oligoribonucleotide utr-c + utr-z (41 mer: 5′-GCAGCGGCGGCGGCAGUGGCGGCGGCGAAGGUGGCGGCGGC-3′) was allowed to adopt its structure in 50 mM Tris–HCl (pH 7.4) and 100 mM KCl (heated to 95°C for 5 min and annealed at RT). For duplex formation, the Cy5.5 oligoribonucleotide utr-c + utr-z was incubated with the core domain of the long noncoding RNA (lncRNA) HSALNT0012722. Three hundred nanograms of double-stranded RNA (dsRNA) was digested with 3U ShortCut RNase III (NEB, Ipswich, MA, USA) in the digestion buffer supplied by the manufacturer at 37°C for 20 min. The reaction mixtures were loaded into an 8% TBE (1×) polyacrylamide gel and then run at 120 V for 1 h at 20°C. After the run, the gel was analysed using the Odyssey CLx Imaging System (Li-COR Biosciences, Lincoln, USA) and then stained with EtBr (0.5 μg/ml in distilled water) and digitally captured using GelDoc (Bio-Rad, USA).

### Chromatin immunoprecipitation and sequencing

Chromatin was extracted from Panc-1 cells treated or not with 1 μM PDS for 16 h. The DNA–protein complexes were cross-linked with 1% formaldehyde (Sigma–Aldrich, Germany) in PBS for 15 min at RT. After quenching and washing twice in PBS, cells were collected and then lysed for 10 min with hypotonic lysis buffer (5 mM Pipes, 85 mM KCl, 0.5% NP-40) containing a protease inhibitor cocktail. The pellets were resuspended in radioimmunoprecipitation assay (RIPA) buffer containing 150 mM KCl and sonicated with the Bioruptor-200 (Diagenode, Belgium) for 30 min with 30-s pulses, resulting in an average size of ∼200 bp for genomic DNA fragments. Samples were pre-cleaned and immunoprecipitated with 3 μg BG4 antibody (Merck, MABE917) or the same amount of control antibody (FLAG M2), followed by a 90-min incubation with protein A blocked with bovine serum albumin and salmon sperm DNA (1 μg/μl) at 4°C. Beads and inputs were treated with proteinase K for 3 h at 56°C to degrade the proteins, and cross-linking was reversed at 68°C O/N. Finally, genomic DNA was purified using the Qiaquick PCR purification kit from Qiagen and eluted in 40 μl of water. Two biological replicates were taken from each sample and subjected to library preparation and sequencing. PE150 with at least 30 million reads per sample were obtained after NGS for each library at Genewiz (Germany).

The ChIP-seq libraries were sequenced on the Illumina HiSeq 2000 platform to generate high-throughput paired-end (PE) reads. Quality assessment of the raw sequencing data was performed using the ShortRead package from the R/Bioconductor suite (RRID:SCR_005824), which allowed assessment of read quality scores, base composition and potential adapter contamination. Reads that passed quality control were aligned to the human reference genome (GRCh38, NCBI build) using Bowtie 2 (RRID:SCR_016368), allowing sensitive and efficient mapping of ChIP-enriched fragments.

Peak calling was performed with MACS2 in “sharp” mode to identify regions o significant enrichment compared to the input controls. Genomic loci of interest and ChIP-seq signal traces were visualized using the Integrative Genomics Viewer (IGV, RRID:SCR_011793) to facilitate inspection of peak profiles and chromatin features. The Bedtools suite (RRID:SCR_006646) was used to further characterize the genomic distribution of G4 structures and assess overlaps between datasets. Overlaps were defined as regions sharing at least one nucleotide, and downstream analyses included annotation of G4 peaks in relation to genomic features.

### RNA-G4 immunoprecipitation and sequencing

Panc-1 cells (30 × 10^6^), treated or not with 1 μM PDS for 16 h, were collected and then lysed for 10 min with hypotonic lysis buffer (5 mM Pipes, 85 mM KCl, 0.5% NP-40) containing a protease inhibitor cocktail. Nucleated pellets were discarded, while the cytoplasmic fraction was diluted 1:1 in G4RP buffer [150 mM KCl, 25 mM Tris (pH 7.4), 5 mM EDTA, 0.5 mM DTT, and 0.5% NP-40 in nuclease-free water]. After pre-cleaning, the lysates were incubated with 3 μg BG4 (Merck) antibody and 8 μl Dynabeads Protein G for 6 h at 4°C. After washing, the beads were incubated with 1 ml of Tri-Reagent (Sigma–Aldrich, Germany) to extract the RNA using a phenol/chloroform extraction method. The RNA was treated with DNaseI (Thermo Fisher Scientific) to digest genomic DNA. Concentration, integrity, and purity of RNA were checked using the Agilent 2100 Bioanalyzer (Agilent RNA 6000 Nano Kit). Ten nanograms of RNA was digested with DNaseI (NEB), purified of ribosomal RNA (using the ribo-zero method), and reverse transcribed to obtain complementary DNA (cDNA). The adapters were ligated to the fragmented cDNA after end repair and A-tailing according to the protocol of Illumina and DNBSEQ (MGI-Tech). A total of 16 rounds of PCR amplification were performed to enrich the cDNA fragments. The PCR products were then purified using Ampure XP beads (Agencourt). The amplified DNA was subjected to paired-end 100 bp sequencing at the BGI Genomics facility (BGI Genomics, China) and at Genewiz (Germany). Two biological replicates were processed separately for each sample.

The quality of the raw sequencing data was assessed using FastQC (RRID:SCR_014583). Reads were aligned to the human reference genome (NCBI GRCh38) using Bowtie 2 (RRID:SCR_016368). Peak calling was performed with MACS2 (v2.2.9.1) in paired-end mode (-f BAMPE), using input and IgG (FLAG M2) controls. Coverage tracks were generated with bamCoverage (deepTools) using the following parameters: bamCoverage –outFileFormat bigwig –binSize 50 -p 10 –normalizeUsing RPKM. Heatmaps of RIP-seq data were generated by quantifying normalized read densities across predefined genomic regions (3 kb around peak summits). Read coverage was computed using the computeMatrix function from the deepTools suite 3.5.6 in reference-point mode, and visualized with plotHeatmap. Data normalization was performed using RPKM.

To annotate G4-enriched peaks relative to TSS, BEDTools 2.31.1 (RRID:SCR_006646), and ChIPseeker 1.44.0 (RRID:SCR_016402) were used. Motif discovery was performed using the MEME Suite (RRID:SCR_001783), specifically employing the MEME algorithm in ZOOPS mode (Zero or One Occurrence Per Sequence), with motif lengths set between 6 and 50 nt. Prediction of G4-forming sequences was carried out using the QGRS Mapper (https://bioinformatics.ramapo.edu/QGRS/index.php) with the following parameters: maximum length = 30, minimum G-group size = 2, loop size = 0–36. The GC content (%GC, guanine-cytosine content) of enriched reads was calculated by extracting the nucleotide sequences corresponding to peak regions and computing the proportion of guanine (G) and cytosine (C) bases relative to the total sequence length. This analysis was performed using deepTools (RRID:SCR_016366) (computeGCbias), allowing for the assessment of sequence composition biases in immunoprecipitated RNA fragments. Finally, density plots around enriched peaks were computed using the computeMatrix function from deepTools (RRID:SCR_016366) in reference-point mode, centred on peak summits, followed by visualization with plotProfile.

### Isothermal titration calorimetry (ITC)

The isothermal titration calorimetry experiments were performed using a Microcal PEAQ-ITC instrument (Malvern Panalytical) at 25°C. All samples were thoroughly dialysed with stirring before use in 10 mM KPi buffer containing 50 mM KCl (DTT was excluded). Titrations were performed with the different utr rG4 sequences. For the ITC titrations, the sample cell was filled to capacity with a dilute solution of UP1 at 8 μM and titrated with utr at 70–110 μM. A typical titration involved the injection of 13–19 (0.4–2 μl) aliquots of titrant, with injections at 200 s intervals. The integrated heat data were corrected for heat of dilution and blank effects and the corrected data were fitted to a binding model by nonlinear regression. The binding isotherms were sigmoidal and fitted well with the standard 0.5 and one-binding site model built into the Microcal PEAQ ITC software. The best fits were always obtained for one-site binding models.

### Production of UP1 and MR spectroscopy

The protein was produced as previously described [30]. The recombinant GST-tagged UP1 protein was expressed in *E. coli* BL21 with the plasmid pGEX-Up1. After transformation, the bacteria were grown overnight in Luria Bertani (LB) medium (10 g/l yeast extract, 10 g/l tryptone, 10 g/l NaCl) with 100 μg/ml ampicillin at 37°C. Cultures were transferred to Terrific Broth and grown to A600 ≥ 2.0 before IPTG induction (1 mM). Post- induction, cells were incubated overnight at 25°C, harvested by centrifugation (5100 rpm, 4°C) and the pellet was resuspended in PBS containing protease inhibitor cocktail (Roche: cOmplete, EDTA-free) and 1 mM DTT. The bacteria were lysed by sonication (40%, 45 s ON/OFF cycles for 4.5 min), followed by centrifugation (35 min, 40 000 rpm, 4°C). The supernatant was incubated with Glutathione-Sepharose 4B beads (GE Healthcare, 50% slurry in PBS) for 30 min at 4°C. The bound proteins were washed five times with PBS and eluted with 20 mM reduced glutathione in 50 mM Tris–HCl (pH 8). To remove the GST tag, the beads were incubated for 4 h at 4°C with PreScission cleavage buffer and protease, which separated UP1 from the GST bound to the beads. Unlabelled UP1 was recovered from the supernatant after centrifugation (500 × *g*, 5 min, 4°C). The purity of UP1 was confirmed by SDS–PAGE

The NMR spectra were recorded using a Bruker AVANCE NEO 700 MHz spectrometer equipped with TXI probe. Water signal was suppressed using excitation sculpting with pulse Sinc1.1000 (2 ms) with a power of 24.8 dB, a bandwidth of 500 Hz combined with gradient pulses. Experiments were generally performed at 25°C, 37°C, and 47°C. All experiments were performed in standard 3 mm NMR tubes containing 8 μl of D_2_O, for a total of 170 μl. The samples were prepared in 10 mM KPi buffer. The concentration of the DNA samples was between 0.1 and 0.3 mM.

### Statistics

The Student’s *t*-test was used for the experimental data. *P* < 0.05 was selected as the statistical significance limit. For comparisons between more than two samples, the one-way analysis of variance (ANOVA) test was used in conjunction with the Kruskal–Wallis test and the Dunn’s test for multiple comparisons. For the correlation between two groups, the Pearson correlation, the Spearman’s correlation, or the Wilcoxon signed-rank test for normal or non-normal distributions were calculated. Excel and GraphPad Prism were used for routine analyses, R/Bioconductor packages for analysing large amounts of data and creating heat maps. We labelled with *P < 0.05, **P < 0.01, ***P < 0.001. Unless otherwise stated, all data in the figures were presented as arithmetic means ± the SDs of at least three independent experiments.

## Results and discussion

### Formation of multiple rG4 structures in the 5′UTR of *KRAS* mRNA

The 5′UTR of *KRAS* mRNA spans 193 nt and contains 33 GG repeats that incorporate rG4 motifs. These motifs can fold into rG4 structures stabilized by two stacked G-tetrads [28, 32] (Fig. 1A and Supplementary Fig. S1). Using the QGRS Mapper tool, three high G-score, nonoverlapping rG4 motifs were identified near the 5′ cap, designated as utr-1, utr-z, and utr-c [28]. CD spectroscopy confirmed that each individual motif assumes a rG4 structure with a parallel topology, as indicated by a strong positive ellipticity at 263 nm and a negative ellipticity at 240 nm [28, 33]. CD melting analyses showed cooperative melting behaviour with melting temperatures (*T*_M_) of 53°C (utr-1), 64°C (utr-z), and 52°C (utr-c) in 50 mM Tris–HCl (pH 7.4) and 100 mM KCl [28].

Additional confirmation of rG4 formation in the 5′UTR was obtained by ¹H NMR experiments (Fig. 1B), which showed for each 5′UTR motif a high ratio of peaks in the imino fingerprint region characteristic of G4s (10–12.5 ppm) compared to the aromatic region (7–9 ppm). Notably, utr-1 and utr-z exhibited less-defined imino patterns, consistent with parallel rG4s, which show spectral broadening due to multiple conformations (particularly in the case of utr-z). Upon heating to 46°C, the spectra of utr-1 became more defined. In contrast, utr-c showed a sharp and distinct imino fingerprint with eight Hoogsteen peaks, suggesting a stable, single rG4 conformer stabilized by two G tetrads (Supplementary Fig. S2).

To determine whether multiple rG4 structures can form simultaneously within the same 5′UTR sequence, we used two complementary approaches. First, we analysed the first 80 nt of the 5′UTR sequence, encompassing all three predicted rG4 motifs (hereafter referred to as s-80), using CD spectroscopy. The s-80 sequence exhibited a strong ellipticity (∼19 mdeg at 265 nm) at a strand concentration of 3 μM (Fig. 1C), which closely matched the cumulative ellipticity (∼21 mdeg) of the three individual rG4 elements. This suggests that at least two rG4 structures should coexist within the s-80 sequence. The presence of rG4 structures within s-80 was also confirmed by the G4-specific antibody BG4 [28].

Next, we asked whether these multiple rG4 structures also form *in vivo*. As shown in Fig. 1D, we generated a series of *KRAS* 5′UTR deletion mutants and constructed lentiviral vectors with wild-type or mutant 5′UTRs fused to the fluorescent protein mScarlet. These included: *pLV-KRAS-5′UTR mScarlet* (with wild-type *KRAS* 5′UTR), *pLV-KRAS-5′UTRmut mScarlet* (with all three rG4 motifs of the 5′UTR disrupted by point mutations; see Supplementary Fig. S3) and various deletion mutants lacking one or two rG4 motifs as illustrated in Fig. 1D. These constructs were stably integrated into the genome of 293T cells, and RIP assays were performed with the BG4 antibody. The recovered RNA was reverse transcribed and amplified with mScarlet-specific primers, using GAPDH-specific primers as a control. G4 enrichment was subsequently quantified (Fig. 1E and Supplementary Table S1). The wild-type 5′UTR construct showed the highest G4 enrichment, whereas the triple mutant (*pLV-KRAS-5′UTRmut* mScarlet) showed negligible G4 enrichment, as expected. Constructs containing a single rG4 motif showed ∼43% of the enrichment observed with wild-type 5′UTR, while those containing any two rG4 motifs showed enrichment nearly equal to that of wild type. Taken together, these results suggest that under physiological conditions, at least two rG4 structures within the 5′UTR of *KRAS* are stably folded, while the third likely exists in a dynamic equilibrium between folded and unfolded states.

### Analysis of the human transcriptome indicates the formation of rG4 structures within the 5′UTR of *KRAS* mRNA

**Figure 2.**
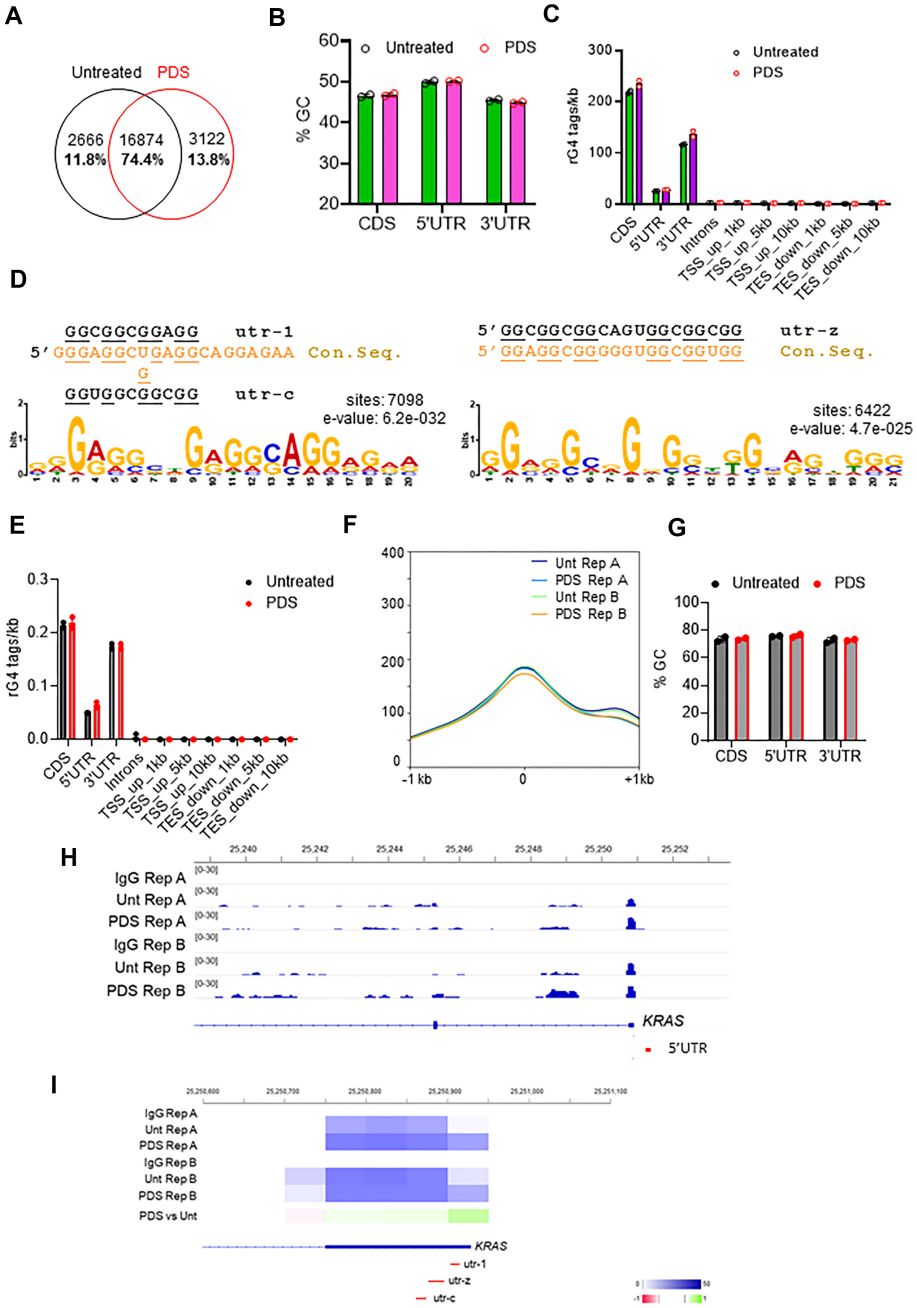
RIP-seq confirms the formation of rG4 structures in the 5′UTR of *KRAS* mRNA. (**A**) Venn diagram showing the number and percentage of rG4 peaks in untreated and PDS-treated Panc-1 cells. (**B**) % GC in FASTA files of the above 16874 rG4 peaks found enriched in both treated and untreated Panc-1 cells, distributed in CDS, 5′UTR and 3′UTR of *KRAS* mRNA. (**C**) Histogram indicating the rG4 tags/kb in the indicated RNA positions in untreated and PDS-treated cells. (**D**) The MEME Suite (RRID:SCR_001783) identified two G-rich consensus motifs among the 16 874 enriched peaks. One motif closely resembles the utr-z sequence, while the other aligns with the utr-1 and utr-c motifs. For each motif, the corresponding WebLogo, enrichment score, and number of occurrences are presented, along with their sequence similarity to the known utr-1, utr-c, and utr-z elements. (**E**) Histogram indicating the rG4 tags/kb in the indicated RNA positions in untreated and PDS-treated cells; Reads were filtered to retain only those matching the enriched motifs shown in Fig. 2D. (**F**) Profile of rG4 signal 2kb around the peaks enriched in both untreated and PDS-treated Panc-1 displaying an optimal rG4 consensus. (**G**) % GC in FASTA files of the above rG4 peaks. (**H**) RIP-seq profiles of Bigwig coverage RPKM-normalized files showing RG4 peaks in the 5′UTR of *KRAS* in untreated and PDS-treated Panc-1 cells. (**I**) Heatmap of RPKM-normalized *KRAS*-rG4 signal (0–50 max) and of the log_2_ratio of rG4 signal between PDS and Untreated samples (−1 to +1). Log_2_ratio was calculated with bigwigcompare 3.5.4 (deeptools2, pseudocount = 11, binSize = 50). Sequence of 5′UTR and positions of utr rG4 motifs are shown. In Fig. 2B, C, E, and G data are shown as mean ± SD. (*n* = 2; two independent pools with three biological replicates.)

The presence of the rG4 structures in the 5′UTR of *KRAS* mRNA under native conditions, i.e. bound to endogenous proteins, was also determined by RNA immunoprecipitation sequencing (RIP-seq) using BG4. Panc-1 cells, untreated or treated for 16 h with 1 μM PDS, a G4 stabilizer, were subjected to RIP with BG4 antibody. Purified RNAs [34], were reverse transcribed into cDNA and analysed by next-generation sequencing. Biological replicates showed high concordance under both untreated and PDS-treated conditions (Supplementary Fig. S4A). More than 70% of the rG4 peaks were conserved under both conditions (Fig. 2A and Supplementary Fig. S4B). PDS treatment induced local enrichments, revealing 13.8% additional peaks compared to untreated cells (Fig. 2A). Analysis of the distribution profiles for the 16 874 enriched peaks under the two conditions showed minimal interference from PDS treatment, confirming that recognition of rG4 domains by the BG4 antibody was not biased by competition with PDS (Supplementary Fig. S4C). The specificity of rG4 immunopurification was further confirmed by a fingerprint plot (Supplementary Fig. S5), which showed that BG4-immunopurified transcripts had higher GC content (47%–51%) than the human genome average (41%), with enrichment being particularly pronounced in the 5′UTR (Fig. 2B). Distribution analysis relative to TSS and transcription end sites showed a bimodal enrichment of rG4 structures at the 5′UTR and 3′UTR (Supplementary Fig. S6). PDS treatment slightly altered this profile by increasing rG4 enrichment within the coding sequences (CDS) and the 3′UTR (Fig. 2C). Motif Discovery identified two G-rich consensus sequences among the 16 874 enriched peaks, one of which showed high similarity to the utr-z motif, while the other consensus sequence matched to utr-1/utr-c (Fig. 2D). Both consensus sequences showed a high propensity to fold into rG4 structures according to QGRS Mapper.

To further refine our analysis, we focused exclusively on the peaks exhibiting enrichment for canonical rG4 structures. These peaks were predominantly localized within CDS, 3′UTRs, and 5′UTRs (Fig. 2E). The distribution patterns were largely consistent between untreated and PDS-treated samples, with the notable exception of the 5′UTR, which showed a marked increase in enrichment following PDS treatment (Fig. 2E, F). As anticipated, these peaks displayed a pronounced GC bias, characterized by a high GC content (Fig. 2G). *KRAS* was identified among the transcripts enriched with rG4 structures.

Notably, genome-wide analysis revealed distinct peaks within the first 80 nt of the 5′UTR of *KRAS mRNA*, consistent with rG4 formation in a native cellular context (Fig. 2H). Heatmaps of rG4 counts confirmed rG4 formation *in vivo* within the utr-c and utr-z regions, whereas utr-1 showed rG4 formation only after PDS treatment, suggesting a lower propensity to fold in the cellular environment (Fig. 2I). Taken together, the RIP-seq results provide strong evidence for rG4 formation within the 5′UTR of *KRAS* mRNA in Panc-1 cells.

### G4 structures both in the promoter and 5′UTR of the *KRAS* gene

**Figure 3.**
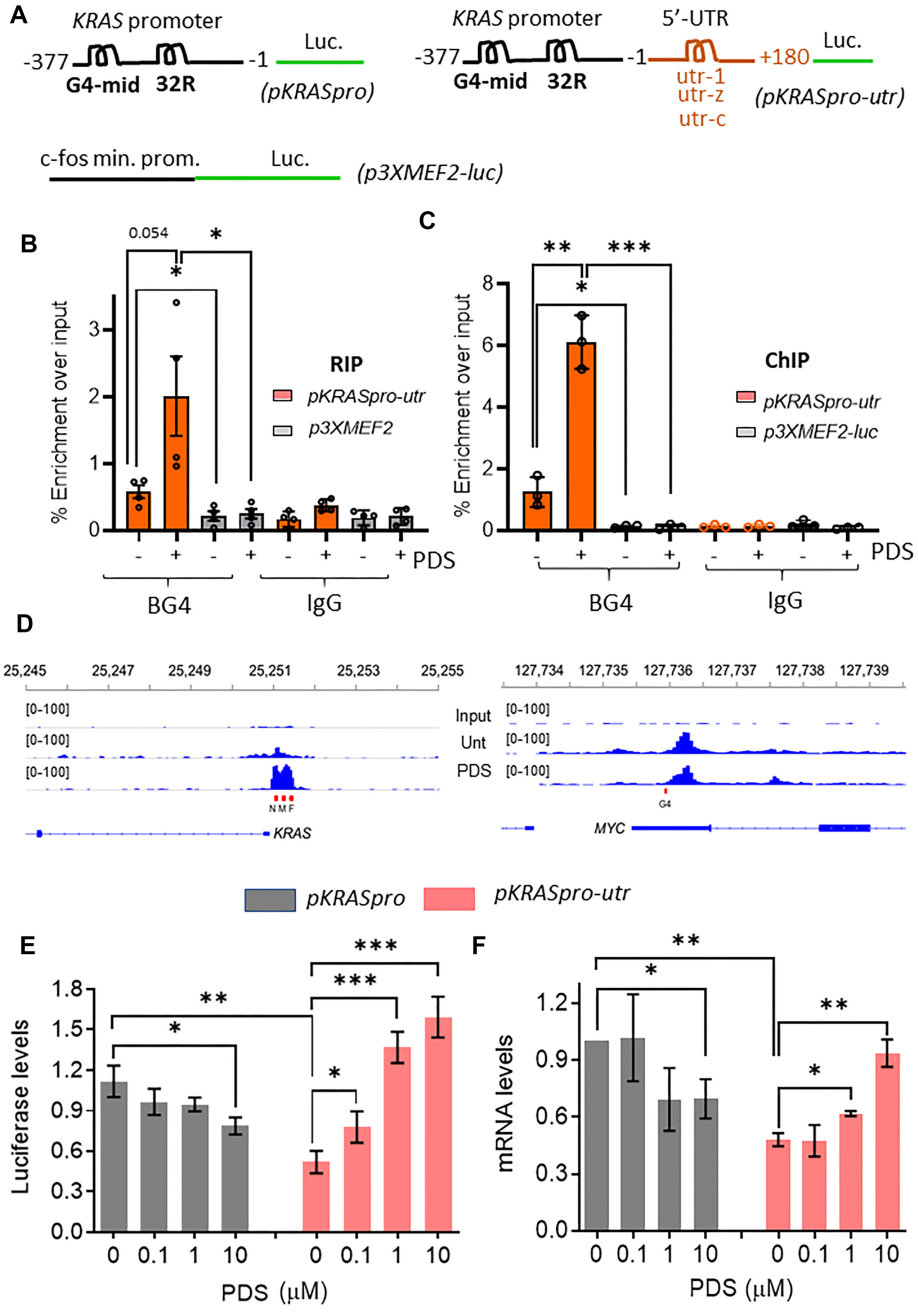
RIP, ChIP, ChIP-seq and effect of 5′UTR on transcription and protein levels. (**A**) The luciferase plasmids *pKRASpro* and *pKRASpro-utr* were developed to study the effects of the 5′UTR on transcription. In *pKRASpro*, luciferase expression is driven by the KRAS promoter (−377/−1 compared to TSS), whereas in *pKRASpro-utr*, luciferase expression is driven by the KRAS promoter (−377/−1) and the 5′UTR of *KRAS* (+1/+180) containing the rG4 motifs utr-1, utr-z, and utr-c. The plasmid *p3XMEF2-luc*, in which luciferase is driven by is driven by three tandem repeats of the MEF2 binding site [CTA(TATT)_4_TAG], positioned upstream of a minimal c-fos promoter, served as a control. (**B**, **C**) RIP (**B**) and ChIP (**C**) assays with the G4-specific antibody BG4 showing the enrichment of rG4 and G4 DNA motifs compared to input in 293T cells transfected with *pKRASpro-utr* or *p3XMEF2-luc* (control), as indicated. The concentration and time of PDS treatment are 1 μM, 24 h. Data are expressed as mean ± SD, *n* = 3 (independent experiments), a *t*-test was performed for the indicated comparisons. (**D**) ChIP-seq shows genomic coverage of DNA sequences after BG4 purification mapped within the *KRAS* and *MYC* genomic loci. (**E**, **F**) Luciferase (left) and mRNA levels (right) in 293T cells transfected with *pKRASpro* or *pKRASpro-utr* and treated or not with the indicated μM concentrations of PDS for 16 h. Data are expressed as mean ± SD, *n* = 4, independent experiments. Dunn’s multiple comparison test was performed.

To investigate the effects of the G4 structures within the *KRAS* gene at the transcriptional and post-transcriptional levels, we constructed three luciferase reporter plasmids using pGL3: (i) *pKRASpro*, which contains the *KRAS* promoter region from −377 to −1 relative to the TSS, including the G4 motifs 32R and G4-mid [35, 36]; (ii) *pKRASpro-utr*, which contains the −377/−1 *KRAS* promoter sequence along with the 5′UTR (+1 to +180) with the rG4 motifs utr-1, utr-z, and utr-c; (iii) *p3XMEF2-luc*, a control plasmid with a minimal c-fos promoter and a MEF2 binding site (an AT-rich, non-G4-forming region) upstream of the luciferase (Fig. 3A).

The formation of rG4 by pKRASpro-utr was detected by RIP with BG4 on lysates from 293T cells transfected with either pKRASpro-utr or control pX3MEF-luc and quantified by RT-qPCR using luciferase-specific primers (Supplementary Table S1 and Fig. 3B). Transfection with pKRASpro-utr resulted in a significant enrichment of luciferase RNA in BG4 pull-downs compared to control p3XMEF2-luc or IgG (*P* < 0.05). In particular, treatment with PDS increased RNA enrichment fourfold (*P* < 0.05). No enrichment was observed in either the p3XMEF2-luc control samples or the IgG samples, suggesting rG4 formation in the 5′UTR of KRAS mRNA under physiological conditions.

To prove G4 DNA formation in the *KRAS* promoter, we performed a ChIP with 293T cells transfected with *pKRASpro-utr*. The extracted DNA was incubated with BG4 and luciferase-specific sequences were quantified by qPCR after treatment with RNase A (Fig. 3C). A fivefold enrichment of the *KRAS* promoter region was observed in BG4 pull-downs compared to input DNA, which increased sixfold after PDS treatment. These results confirm G4 formation in the *KRAS* promoter, which is consistent with previous studies [24, 25].

To validate G4 DNA formation in a more physiologically relevant model, we performed a BG4 ChIP-seq in Panc-1 cells. Significantly enriched peaks were detected at the *KRAS* promoter in the region corresponding to the 32R and G4-mid motifs, which were markedly enhanced by PDS treatment (Fig. 3D) [24, 35]. As an additional control, we identified a significant G4 peak corresponding to the well-characterized parallel G4 structure within the *MYC* promoter region (pdb_00001xav).

Overall, our RIP, ChIP, RIP-seq, and ChIP-seq results provide convincing evidence for the *in vivo*formation of G4 structures within the KRAS gene in its promoter and 5′UTR. To investigate the effects of promoter G4 DNA and 5′UTR rG4s on KRAS expression, we performed luciferase assays in 293T cells transfected with either plasmid pKRASpro or plasmid pKRASpro-utr and treated with increasing concentrations of PDS (0, 0.1, 1, and 10 μM) (Fig. 3E). The results of the dual luciferase assay showed that: (i) incorporation of the 5′UTR downstream of the KRAS promoter (−377/−1) resulted in a ∼50% reduction in luciferase activity (*P* = 0.0014); (ii) cells transfected with pKRASpro and treated with PDS showed a dose-dependent decrease in luciferase levels, consistent with the fact that PDS targets guanine clusters that tend to fold into G4 and modulate gene expression [37]; (iii) cells transfected with pKRASpro-utr instead showed a significant, dose-dependent increase in luciferase levels after treatment with PDS. This indicates that the stabilization of 5′UTR rG4 structures leads to an increase in luciferase levels: an effect that overrides the effect observed with pKRASpro and underlines the crucial role of 5′UTR in *KRAS*regulation. We also measured luciferase mRNA levels and observed a strong correlation with the corresponding luciferase protein expression. Consistent with luciferase protein data, 293T cells transfected with *pKRASpro-utr* exhibited a 50% reduction in luciferase mRNA levels compared to cells transfected with *pKRASpro* (*P* < 0.01) (Fig. 3F). In addition, treatment with PDS produced effects on luciferase mRNA levels similar to those seen when the protein levels were measured. In pKRASpro-transfected cells, PDS induced a dose-dependent decrease in luciferase mRNA levels. In contrast, PDS treatment in pKRASpro-utr-transfected cells resulted in a twofold increase in mRNA levels.

### The 5′UTR reduces the stability of *KRAS* mRNA

**Figure 4.**
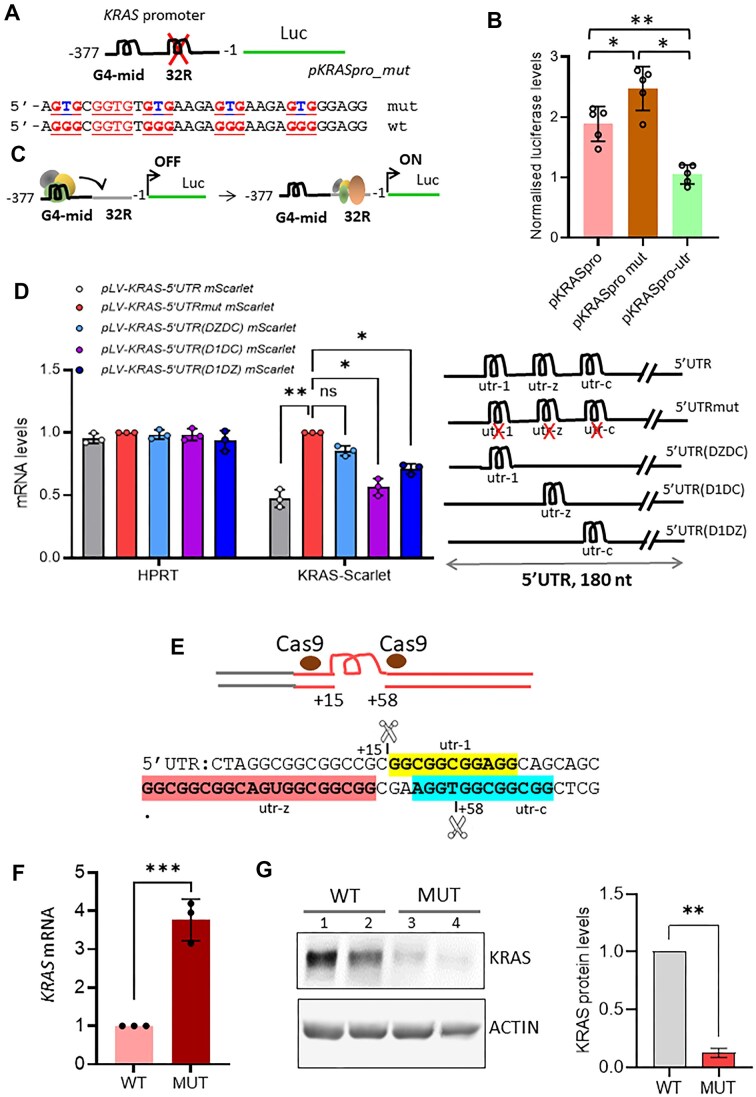
Influence on *KRAS* gene expression of G4 and rG4 in the promoter and 5′UTR mRNA, respectively. (**A**) Schematic representation of plasmid *pKRASpro_mut* containing upstream of luciferase the *KRAS* promoter with mutations in the 32R motif that destabilise G4 formation. (**B**) Relative luciferase levels in 293T cells transfected with 1 μg of the indicated plasmids and 100 ng of pRenilla, as an internal reference. Data are expressed as mean ± SD, *n* = 4, independent experiments. Dunn’s multiple comparison test. (**C**) Putative representation of G4-mid as a recruitment element for TFs, which then bind to the neighbouring 32R motif in the linear duplex conformation. (**D**) Levels of *mScarlet* and *HPRT* mRNAs measured in 293T cells stably transfected with the indicated lentiviral plasmids carrying either the wild-type KRAS-5′UTR or mutant KRAS-5′UTRmut in which utr-1, utr-c, and utr-z motifs are mutated or KRAS-5′UTR with deletion mutations carrying only one rG4 domain as shown in the scheme. Data are expressed as mean ± SD, *n* = 3, independent experiments. Dunn’s multiple comparison test. (**E**) Cas9 was used to remove utr-1, utr-z, and part of utr-c from KRAS-5′UTR in 293T cells. (**F**) *KRAS* mRNA levels relative to *HPRT* in wild-type and mutant (MUT) 293T cells, *n* = 3, independent experiments, *t*-test was performed. (**G**) Immunoblot analysis of KRAS protein levels in wild-type and mutant 293T cells (2 biological replicates for each clone). Right panel shows the quantification of the KRAS protein; a *t*-test was performed.

To explain the observed increase in mRNA levels after PDS treatment, we hypothesized that PDS stabilizes rG4 structures in the 5′UTR and thereby increases *KRAS* mRNA stability. To test this mechanism, we introduced point mutations into the *KRAS* promoter at the 32R motif and point mutations and/or deletions within the *KRAS*5′UTR to disrupt G4 formation. First, we constructed a reporter plasmid, namely *pKRASpro_mut*, in which four Gs were replaced by Ts in G runs 1, 3, 4, and 5 of the 32R motif located in the *KRAS* promoter upstream of the TSS and recognized by the TFs PARP1, MAZ, and hnRNPA1 [12, 24, 25] (Fig. 4A). Luciferase assays showed that mutations in the 32R motif significantly increased luciferase activity compared to cells transfected with the wild-type *pKRASpro* vector (*P*< 0.05) (Fig. 4B). To explain this transcriptional effect, we propose that the G4-mid structure, located ∼1.5 helical turns upstream of 32R, serves as a recruitment platform for TFs. Their subsequent incorporation into the transcriptional machinery at the neighbouring 32R site should be facilitated by the double-stranded conformation of the mutated site [24] (Fig. 4C).

Inclusion of the 5′UTR in the vector reduced luciferase expression by ∼50% (*P* < 0.01), indicating that the 5′UTR plays a central role in regulating mRNA levels. To further dissect this effect, we analysed the individual contributions of each rG4 motif within 5′UTR. We generated a series of deletion mutants, each containing only one rG4 element (as shown in Fig. 1D), and stably transfected them into 293T cells. mScarlet mRNA levels were then quantified by RT-qPCR. As shown in Fig. 4D, cells transfected with the full-length *pLV-KRAS-5′UTR mScarlet* construct had the lowest mRNA levels, whereas *pLV-KRAS-5′UTRmut mScarlet*, in which all rG4 motifs were mutated, had the highest mRNA levels. Constructs containing a single rG4 motif showed different regulatory contributions of the individual elements. Deletion of utr-z and utr-c, leaving only utr-1 [5′UTR(DZDC)], resulted in a significant increase in transcript levels compared to wild-type 5′UTR, almost reaching the levels of the mutant construct. This indicates that utr-z and utr-c contribute substantially to the down-regulation of transcripts, whereas utr-1 has a relatively minor effect. In contrast, deletion of utr-1 and utr-c, leaving only utr-z [5′UTR(D1DC)], resulted in no significant change in mRNA levels compared to the wild-type construct, emphasising that utr-z is the major regulatory element controlling mRNA levels. When utr-1 and utr-z were deleted and only utr-c remained [5′UTR(D1DZ)], the transcript levels were in between, indicating a moderate role of utr-c in determining mRNA levels.

Overall, these results indicate a graded contribution of rG4 motifs to the regulation of mRNA levels, following the pattern: utr-z > utr-c > utr-1. A similar trend was observed at the protein level, as evidenced by mScarlet expression (Supplementary Fig. S7). These findings suggest that a complex regulatory mechanism involving rG4 motifs plays a central role in controlling the stability of the *KRAS* transcript.

### The mechanism of mRNA stability involves 5′UTR rG4 structures and lncRNAs

An insight into the mechanism regulating the stability of *KRAS* mRNA was obtained by CRISPR/Cas9 which was used to delete 43 nt, from +15 to +58, in the *KRAS* 5′UTR in 293T cells (Fig. 4E). The deleted segment included the G4 motifs utr-c, utr-z, and four bases of utr-1. We measured *KRAS* mRNA by RT-qPCR and KRAS protein by western blot in both wild-type and CRISPR/Cas9-edited cells (called MUT cells) (Fig. 4F and G). Deletion of the rG4 motifs in the *KRAS* 5′UTR resulted in a fourfold increase in mRNA (*P* < 0.001) consistent with the observation that 5′UTR reduces the mRNA stability (Fig. 4F). Remarkably, we found that the 5′UTR deletion led to a strong downregulation of KRAS protein (Fig. 4G). A similar unusual behaviour was observed in H9C2 cells, where Nkx2-5 mRNA levels increased and Nkx2-5 protein levels decreased in cells where RHAU was silenced. Nie *et al.* [38] found that RHAU, a helicase associated with the 5′UTR and 3′UTRs of Nkx2-5 mRNA, determines the half-life of Nkx2-5 mRNA: overexpression of RHAU shortened the half-life, whereas knockdown of RHAU prolonged the half-life. To explain this discrepancy between transcript and protein content, it is important to remember that the deletion at the 5′UTR can impair the scanning of the smaller subunit of the ribosome, resulting in reduced translation, similar to what we have previously reported [28].

**Figure 5.**
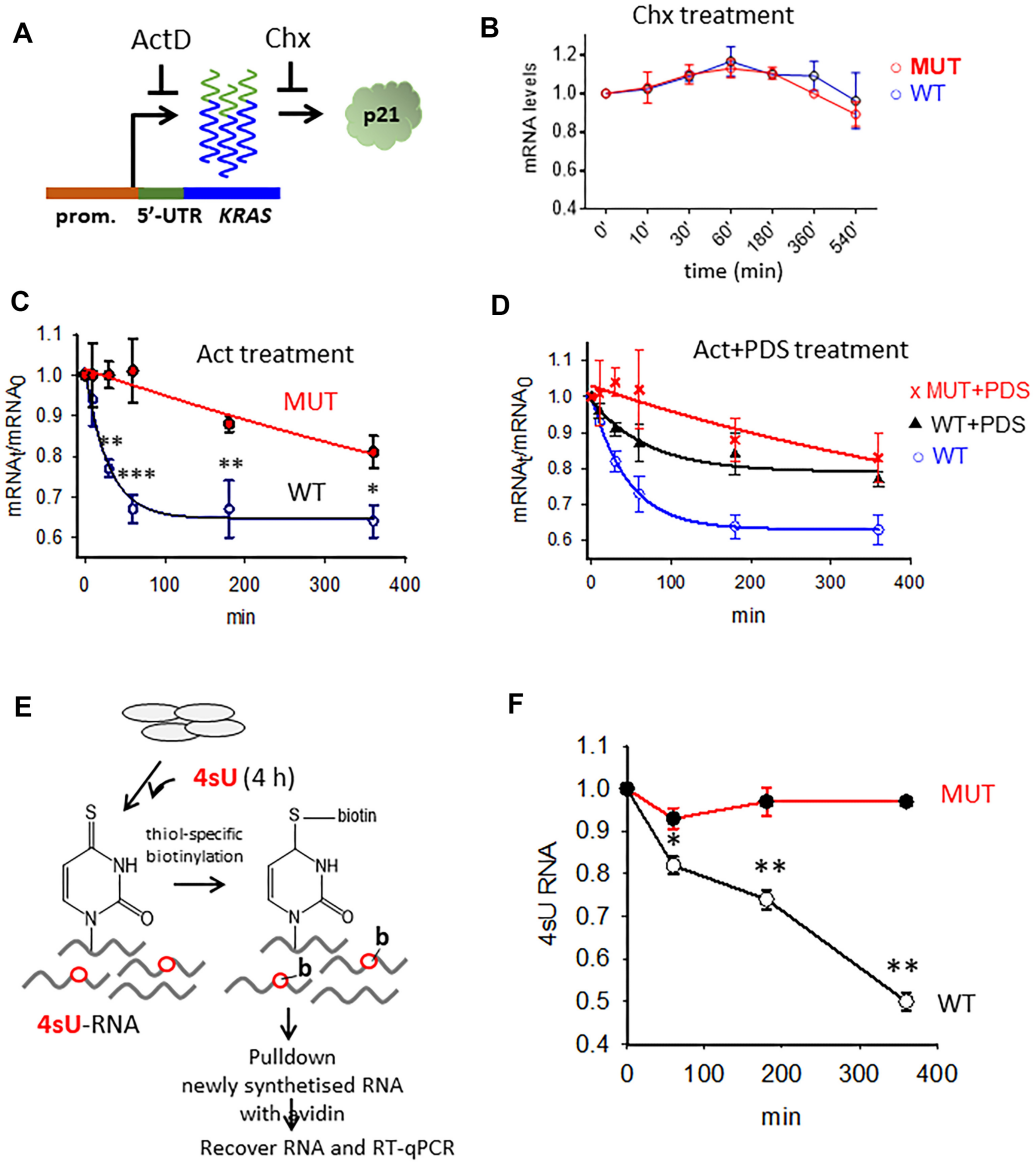
Stability of *KRAS* mRNA and effect of lncRNA. (**A**) Scheme of the experiment for monitoring the effect of the 5′UTR of *KRAS* on mRNA stability. (**B**–**D**) Wild-type (WT) and CRISPR/Cas9 edited 293T cells (MUT cells, in which the rG4 motifs in 5′UTR were deleted) were treated or not with 1 μM PDS for 12 h and then with 10 μg/ml Chx or with 20 nM ActD (**C**, **D**). The cells were harvested at the indicated time points after treatment. Experimental mRNAt/mRNA_0_ plots were best fitted to a standard first-order decay equation. *n* > 3, independent experiments, *t*-test was performed. (**E**) Pulse-chase labelling of newly synthesized mRNA with 4sU in wild-type and MUT 293T cells. The cells were treated with 4sU for 4 h and subject to thiol-specific biotinylation with Biotin-HPDP. Newly synthesized RNA was pulldown with streptavidin beads, recovered and subjected to RT-qPCR. (**F**) WT and MUT 293T cells were treated with 4sU for 4 h and mRNA determined as a function of time by RT-qPCR. Data are expressed as mean ± SD, *n* = 3, independent experiments, *t*-test.

We therefore investigated whether and how the 5′UTR actually controls the stability of *KRAS* mRNA. To exclude nonsense-mediated decay (NMD) due to the presence of a short upstream open reading frame in the 5′UTR or 5′UTR-controlled changes in the splicing profile, we treated both wild-type and MUT 293T cells with Chx, an inhibitor of protein synthesis in eukaryotes [39], and monitored *KRAS* mRNA levels over time by RT-qPCR (Fig. 5A and B). Remarkably, levels of *KRAS* transcripts remained comparable in both wild-type and MUT cells for up to 540 min, indicating that the 5′UTR does not control NMD-like mechanisms.

Next, using ActD, a DNA-intercalating molecule that blocks transcription [40], we examined the time course of mRNA levels (Fig. 5C). The results showed that wild-type 293T cells with the rG4 motifs in the *KRAS* 5′UTR had significantly lower *KRAS* mRNA levels after ActD treatment than MUT cells lacking the rG4 motifs in the 5′UTR, at all time points between 0 and 380 min. The decrease in mRNA occurs within the first 60 min (kinetic part of the curve). At longer time periods, the experimental points lie in a plateau indicating equilibrium. By plotting ln *r* versus time relative to the kinetic curve, where *r* = [RNA]_t_/[RNA]_0_, [RNA]_t_ and [RNA]_0_ are the mRNA concentrations at time *t* and 0, respectively, we obtained a straight line indicating a first-order kinetics for the mRNA decline (Supplementary Fig. S8). We then fitted all experimental points to a first-order kinetic equation and obtained a kinetic constant k = 3.5 × 10^−2^ ± 7.4 × 10^−3^ min^−1^ and a half-life t$\frac{1}{2}$ of 0.32 h (t$\frac{1}{2}$ = 0.693/k). In contrast, the transcript levels in the MUT cells lacking the rG4 motifs gave a k = 1.2 × 10^−3^ ± 2.6 × 10^−3^ min^−1^ and a half-life of 9.6 h. These data show that the wild-type *KRAS* mRNA has a 30-fold shorter half-life than the mRNA from the MUT cells, clearly indicating that the 5′UTR affects mRNA stability.

The wild-type and MUT 293T cells were also treated with ActD alone or in combination with PDS, and *KRAS* mRNA was monitored over time (Fig. 5D). The effect of PDS was to reduce the rate of mRNA decay, with the kinetic constant k decreasing from 3.5 × 10^−2^ ± 7.4 × 10^−3^ min^−1^ to 1.1 × 10^−2^ ± 4.6 × 10^-3^ min^−1^ and the half-life decreasing by 3.3-fold, from 0.32 to 1.05 h. As expected, the MUT cells showed no effect of PDS on *KRAS* mRNA decay: the half-life of *KRAS* mRNA varied between 9.6 h (without PDS) and 8.9 h (in the presence of PDS).

Since transcription, translation and mRNA stability are closely linked, ActD could interfere with these important cellular processes. Therefore, we tracked mRNA levels under more natural conditions using a metabolic pulse-chase labelling approach (Fig. 5E). Specifically, we labelled nascent RNA with 4sU, which allowed us to distinguish between newly synthesised and pre-existing mRNA in the cell [41]. The results shown in Fig. 5F demonstrate a reduction in mRNA in wild-type 5′UTR cells, but not in MUT cells lacking the rG4 motifs. These results provide further evidence that the 5′UTR plays a central role in determining mRNA stability.

**Figure 6.**
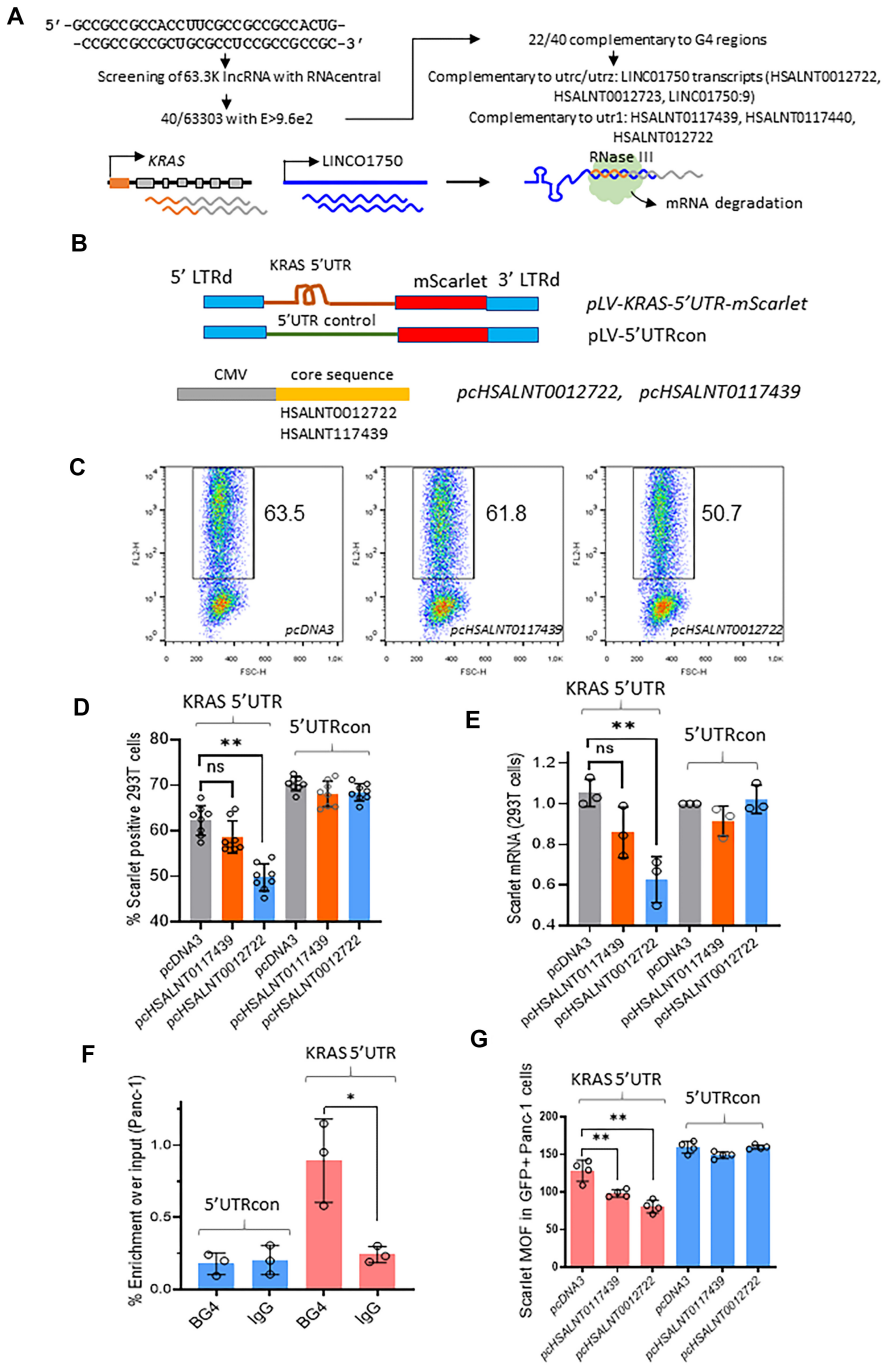
LncRNA HSALNT0012722 promotes the downregulation of *KRAS*. (**A**) RNAcentral v24 was interrogated with the complementary sequence of s-80 to identify lncRNAs containing this motif with the potential to form a duplex with the 5′UTR of *KRAS*. A total of 40 lncRNAs were fished out with a significance threshold of E > 9.6e^2^. A total of 18/40 were selected because they are complementary at the utr-1, utr-c, and utr-z level, as indicated. Four of these (highlighted in the table) were selected because they contain a region that is complementary to utr-c and utr-z (LINC01750.9, HSALNT0012723, and HSALNT0012722) or utr-1 (HSALNT0117439). A schematic representation of the mechanism of action of the lncRNA in regulating optimal levels of *KRAS* mRNA is provided. (**B**) Fluorescent mScarlet was inserted into plasmid pLV under the control of KRAS 5′UTR (pLV-KRAS-5′UTR Scarlet) or control 5′UTR (Addgene plasmid #184637) (*pLV-5′UTRcon*). The core sequences of lncRNA HSALNT0012722 and HSALNT0117439 complementary to utr-z/utr-c and utr-1, respectively, were cloned into *pcDNA3* downstream of the CMV promoter to obtain *pcHSALNT0012722* and *pcHSALNT0117439*. (**C**) The mScarlet vectors were integrated into 293T cells. A total of 1 × 10^6^ 293 founder cells were transfected with 5 μg of the indicated *pcDNA3* plasmids containing the core lncRNA sequence. The % mScarlet positive 293T cells was determined by flow cytometry 48 h after transfection with *pcDNA3*, *pcHSALNT0012722* or *pcHSALNT0117439*. (**D**,**E**) Histograms showing the percentage of mScarlet positive cells (**D**) and mScarlet mRNA levels (**E**) in 293T cells stably transfected with *pLV-KRAS-5′UTR Scarlet* or *pLV-5′UTRcon mScarlet*. Data are expressed as mean ± SD, *n* = 8 in panel (D), *n* = 3 in panel (E), independent experiments. Dunn’s multiple comparison test was performed. (**F**) A RIP assay with BG4 was performed in Panc-1 cells to assess rG4 formation in the reporter by precipitating the RNA bound by BG4 and IgG. To subtract the possible formation of G4 in the mScarlet CDS, the same experiment was performed in cells that had integrated the control 5′UTR. Data are expressed as mean ± SD, *n* = 3, independent experiments. (**G**) A total of 3 × 10^5^ Panc-1 founder cells generated as described in Fig. 6E were co-transfected with 2 μg *pcDNA3* plasmids expressing the indicated lncRNAs and 500 ng pEGFP C1 with 6 μl Lipofectamine 2000. After 48 h, the GFP + cells were scored for mScarlet positivity by flow cytometry. Data are expressed as mean of fluorescence (MOF) in the fraction of GFP + cells. Data are expressed as mean ± SD, *n* = 4, independent experiments. Dunn’s multiple comparison test was performed.

To explain how the 5′UTR controls mRNA stability, we hypothesized that mRNA stability may depend on a mechanism involving lncRNAs. Previous research has emphasized the multiple functions of lncRNAs in regulating gene expression, often through interactions with nucleic acids [42]. Certain lncRNAs, such as lincRNA-p21 and OIP5-AS1, modulate the expression of target RNAs via sequence complementarity [43]. By hybridizing with their target transcripts, these lncRNAs can form double-stranded RNA domains (dsRNA) that are recognized by specific proteins [42–46]. On this basis, we analysed 63,303 lncRNAs from RNAcentral (v24) and identified 40 with sequence complementarity to the 5′UTR of *KRAS* mRNA, particularly in regions containing rG4 motifs. Of these, 22 lncRNAs showed complementarity with at least one of the 3 rG4 motifs (Fig. 6A) applying the following criteria: a maximum of one mismatch allowed in rG4, and a complementarity cut-off score E > 9.6e^2^ by using RNAcentral similarity sequence tool restricted to human lncRNAs. We further refined this list to 18 lncRNAs which were previously reported to be transcribed in human biological samples. In particular, LINC01750:9, LINC01750:12, HSALNT0012722, and HSALNT0012723 transcripts, which originate from the LINC01750 locus, contain domains that show complementarity to the utr-z and/or utr-c motifs (Supplementary Fig. S9). In addition, HSALNT0117439 shows complementarity with the utr-1 motif. These lncRNAs may regulate *KRAS* mRNA levels by base pairing with its 5′UTR, potentially recruiting RNA-degrading enzymes such as RNase III. This endoribonuclease hydrolyzes phosphodiester bonds at dsRNA target sites by activating water as a nucleophile, resulting in cleavage products with 2-nucleotide 3′-overhangs [47–49].

### LncRNAs recognise utr-z and utr-c rG4 motifs and control *KRAS* stability

To support our hypothesis, we developed four expression vectors. We inserted the core sequence (39 nt) of lncRNA HSALNT0012722, which is complementary to utr-z and utr-c, and the core sequence of HSALNT0117439 (42 nt), which is complementary to utr-1 (Supplementary Table S1), into vector *pcDNA3* downstream of the CMV promoter to obtain plasmids *pcHSALNT0012722* and *pcHSALNT0117439* (Fig. 6B). The *pcDNA3* backbone was chosen to allow immediate polyadenylation of the transcribed lncRNAs. In addition, we used the lentiviral vector *pLV-KRAS-5′UTR mScarlet* containing the CDS of the red fluorescent protein mScarlet flanked by the *KRAS* 5′UTR sequence (+1/+179). We also constructed a control vector, *pLV-5′UTRcon mScarlet*, containing CMV 5′UTR upstream of mScarlet [50] (Fig. 6B). The lentiviral vectors were integrated into the genome of human 293T cells, resulting in clones that produce the mScarlet protein under the control of either *KRAS* 5′UTR or 5′UTRcon.

The 293T clones carrying *KRAS* 5′UTR or 5′UTRcon were transfected with either the vectors expressing the core sequence of the lncRNAs, *pcHSALNT0012722* and *pcHSALNT0117439*, or with the control vector *pcDNA3*. We observed that the core sequence HSALNT0012722, which is complementary to utr-c and utr-z, reduced the amount of mScarlet protein by ∼20% (*P* < 0.01), whereas HSALNT0117439, which is complementary to utr-1, by only ∼5% (Fig. 6C and D). In contrast, the 293T clone carrying 5′UTRcon was not affected by lncRNA, as expected. We also quantified mScarlet mRNA in 293T cells stably transduced with *pLV-KRAS-5′UTR mScarlet*. The results showed that *pcHSALNT0012722* reduced mScarlet mRNA levels by ∼40% (*P* < 0.01) compared to the control (*pcDNA3*), while HSALNT0117439 reduced levels by ∼15% (Fig. 6E). The lentiviral mScarlet vectors were also integrated into the genome of Panc-1 cells to investigate the effect of lncRNA core sequences on mScarlet expression in PDAC cells. A RIP assay with BG4 showed that the *pLV-KRAS-5′UTR mScarlet* construct was successfully integrated into the Panc-1 genome (Fig. 6F). Both *pcHSALNT0012722* and *pcHSALNT0117439* reduced the number of cells expressing the mScarlet protein by ∼40% (P = 0.0011) and ∼20% (P = 0.0065), respectively (Fig. 6G). Taken together, these data are consistent with our hypothesis that specific lncRNAs reduce the half-life of *KRAS* mRNA, likely by recruiting RNase III, a ribonuclease that specifically degrades double-stranded RNA [47–49].

### LINC01750 lncRNAs are associated with the *KRAS* 5′UTR and modulate mRNA levels

**Figure 7.**
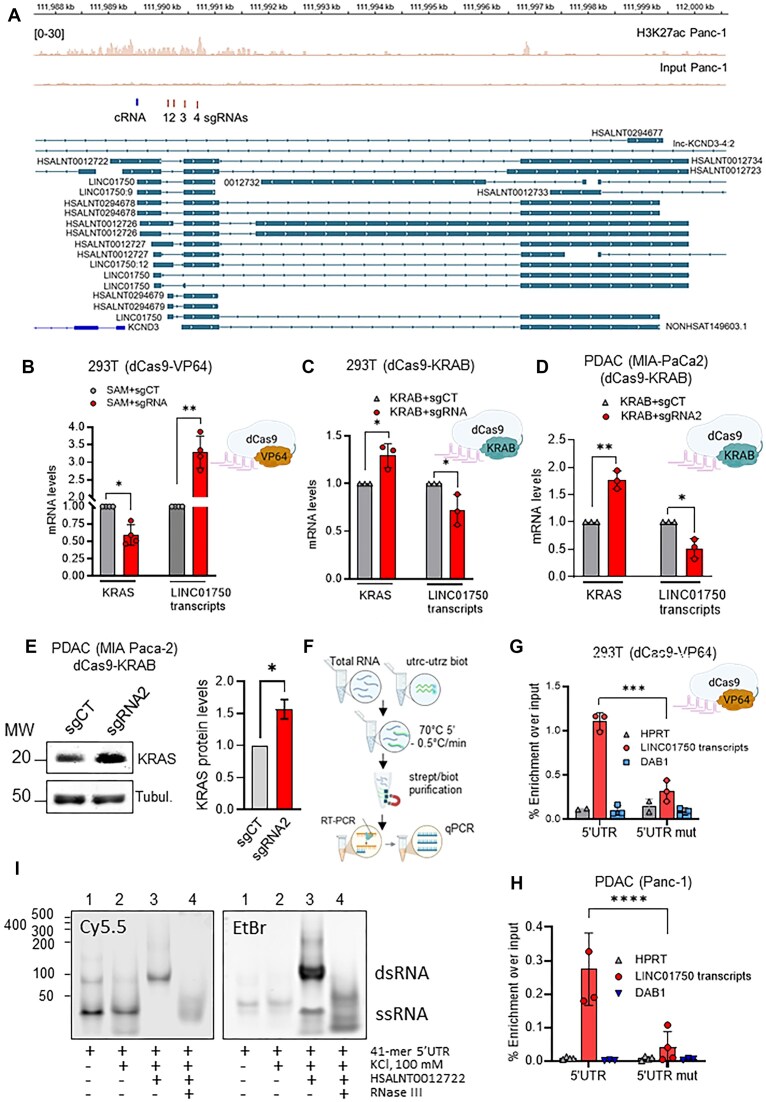
LINC01750 lncRNAs bind to KRAS 5′UTR and affect *KRAS* mRNA levels (**A**) Schematic representation of the locus of LINC01750 transcripts. H3K27ac acetylation in Panc-1 cells compared to input, the positions of the four sgRNAs used to modulate lncRNA expression, and the region complementary to the 5′UTR of KRAS [indicated as complementary RNA (cRNA)] are shown. (**B**) Levels of *KRAS* mRNA and LINC01750 transcripts in 293T cells in which LINC01750 expression was enhanced by the dCas9-VP64 system (SAM + sgRNA) compared to control (SAM + sgRNACT). (**C**) LINC01750 transcripts were repressed in 293T cells by the dCas9-KRAB system (KRAB + sgRNA). As a control, the cells were transduced with KRAB + sgRNACT. (**D**) Levels of *KRAS* mRNA and LINC01750 transcripts in PDAC cells (MIA PaCa-2) in which lncRNA expression was suppressed by the dCas9-KRAB system (KRAB + sgRNA2) compared to control (KRAB + sgRNACT). (**E**) Western blot showing the level of KRAS protein in PDAC (MIA PaCa2) cells transduced with either KRAB + sgRNA2 or KRAB + sgRNACT. The histogram shows the KRAS/Tubulin ratio in the treated cells. (**F**–**H**) Scheme of the Biotin-streptavidin assay using RNA extracted from 293T (transduced with dCas9-VP64 to overexpress LINCO17250) (Fig. 7G) and PDAC (Panc-1) cells (Fig. 7H). Biotinylated 41-mer RNA with the utr-c and utr-z motifs was used as bait, while a 41-mer mutated at the rG4 motifs served as a control. Significant enrichment of LINC01750 transcripts was only achieved with the wild-type bait. No enrichment of HPRT and *DAB1* transcripts was obtained (control). (**I**) PAGE showing RNase III-dependent degradation of KRAS 5′UTR hybridised to LncRNA. Twenty micromolar Cy5.5-labelled oligoribonucleotide containing the utr-z + utr-c motifs (41-mer 5′UTR) was melted at 95°C for 5 min and annealed for 3 h at RT in 50 mM Tris (pH7.4) in the presence or absence of 100 mM KCl. Where indicated, a 39-mer synthetic HSALNT0012722 was added in a 2:1 ratio to the 41-mer 5′UTR to allow formation of dsRNA. dsRNA was digested with 3U RNase III for 15 min at 30°C. The experiment was repeated twice with similar results.

Based on the effects of HSALNT0012722 core expression on Scarlet mRNA levels in 293T cells stably transduced with *pLV-KRAS-5′UTR mScarlet* (Fig. 6E), we examined the effects of modulating the expression of endogenous lncRNAs on *KRAS* transcript levels. The lncRNA HSALNT0012722 is transcribed from the LINC01750 locus along with other lncRNAs that are complementary to the utr-z motif of 5′UTR, namely LINC01750:9, LINC01750:12, and HSALNT0012723 (Fig. 7A and Supplementary Fig. S9). Our previous H3K27ac ChIP-seq analysis [51] showed that this locus resides within an open chromatin region, making it a suitable target for CRISPR-based transcriptional modulation with dCas9 fused to either KRAB (repression) or VP64 (activation) [52], guided by four sgRNAs targeting this accessible region (Fig. 7A). We first used the dCas9-VP64 system (referred to as SAM in Fig. 7) [53] to activate transcription of LINC01750. This approach resulted in >3-fold increase in the expression of LINC01750:9, HSALNT0012722 and HSALNT0012723 (Fig. 7B). Specific primer sets were used for the amplification of these transcripts (Supplementary Table S1). In parallel, an RT-qPCR analysis revealed a ∼50% reduction in *KRAS* mRNA levels compared to 293T cells transfected with control sgRNAs (Fig. 7B). These results suggest that LINC01750 locus-derived lncRNAs negatively regulate endogenous *KRAS* mRNA *in vivo*.

In a complementary set of experiments, we used the dCas9-KRAB system to suppress LINC01750 expression. While this only led to a partial downregulation of its transcripts (∼30%, *P* < 0.05), a corresponding increase in *KRAS* mRNA levels (∼30%, *P* < 0.05) was observed, consistent with the inverse relationship observed with activation (Fig. 7C). Consistent with a previous study, transcriptional activation with SAM is more robust than repression with dCas9-KRAB [54, 55]. However, to increase the efficiency of LINC01750 repression, we tested each of the four sgRNAs individually in PDAC cells and identified the one (sgRNA2; see the ‘Materials and methods’ section) that produced the strongest transcriptional repression in the presence of dCas9-KRAB (Fig. 7D). In PDAC cells (MIA PaCa2) we achieved ∼50% suppression of LINC01750 (*P* < 0.05) and simultaneous upregulation of 75% of *KRAS* mRNA (*P* < 0.01) (Fig. 7D) and KRAS protein by ∼50% (*P* < 0.05) (Fig. 7E).

To confirm the direct interaction between LINC01750-derived lncRNAs (LINC01750:9, HSALNT0012722, and HSALNT0012723) and the 5′UTR of *KRAS* mRNA, we performed streptavidin–biotin RNA pull-down assays (Fig. 7F). Two biotinylated RNA baits were used: one containing the wild-type *KRAS* 5′UTR, which includes the *utr-z* and *utr-c* rG4 motifs, and another containing a mutant version with disrupted rG4 motifs (Supplementary Table S1). Each RNA bait was incubated for 4 h with 100 μg of total RNA extracted either from 293T cells, where LINC01750 expression was upregulated using dCas9-VP64 (Fig. 7G), or from PDAC cells (Panc-1), which express endogenous levels of LINC01750 (Fig. 7H). RNA bound to the baits was recovered using avidin-coated agarose beads, reverse transcribed, and quantified by qPCR. The results demonstrated that LINC01750 transcripts were efficiently captured by the wild-type RNA bait in both cell types, but not by the mutant bait, confirming direct binding of the lncRNAs to the *utr-z* and *utr-c* motifs. No interaction was observed with control RNAs from *HPRT* or *DAB1*.

To further explore the mechanistic basis of lncRNA-mediated *KRAS* regulation, we examined whether HSALNT0012722 facilitates RNase III-dependent degradation of the 5′UTR of *KRASin vitro* (Fig. 7I). A 41-mer oligoribonucleotide corresponding to 5′UTR of *KRAS* containing the rG4 motifs utr-z and utr-c and labelled with Cy5.5 at the 5′-end, was annealed in 100 mM KCl buffer with or without a complementary 39-mer HSALNT0012722 RNA strand (2:1 ratio of lncRNA to RNA target). After duplex formation, the RNA complex was treated with RNase III for 15 min. Samples were run in a polyacrylamide gel and visualized by both Cy5.5 fluorescence and ethidium bromide staining. The results showed that: (i) the 41-mer 5′UTR migrates mainly as a monomer and in small amount as a homodimer stabilized by 21 CG base pairs; (ii) in 100 mM KCl, the 41-mer oligonucleotide run only as a monomer due to rG4 stabilization; (iii) addition of the complementary 39-mer HSALNT0012722 leads to the formation of an RNA duplex, as evidenced by a retarded band; (iv) the RNA duplex is completely degraded by RNase III. The excess HSALNT0012722 in lane 3, visualized by EtBr, is also degraded by RNase III, as it can fold into a hairpin-like structure according to RNA Fold. Overall, these results demonstrate that LINC01750-derived lncRNAs bind directly to the 5′UTR of *KRAS* mRNA and thereby modulate transcript levels, likely by forming an RNA duplex domain that is recognized and degraded by RNase III.

### The 5′UTR rG4 structures of *KRAS* mRNA are bound by hnRNPA1

**Figure 8.**
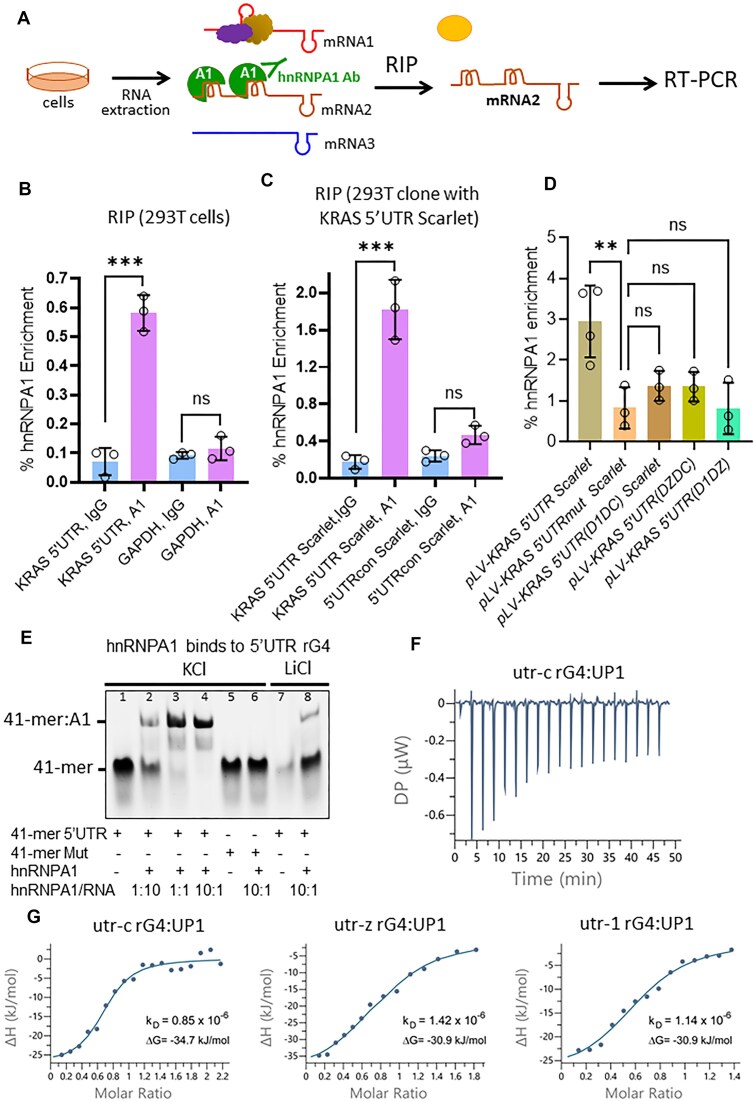
5′UTR rG4 structures are bound by hnRNPA1. (**A**) RIP scheme to show that hnRNPA1 is associated with KRAS 5′UTR containing the rG4 motifs. (**B**) RIP performed with 293T cells using hnRNPA1 Ab and IgG (control) shows that hnRNPA1 is associated with the 5′UTR of *KRAS* mRNA containing the rG4 motifs. As a further control, we show no enrichment of GAPDH. (**C**) Same experiment as in panel (B), but using 293T cells in which *pLV-KRAS-5′UTR mScarlet* or *pLV-KRAS-5′UTRcon mScarlet* were integrated into the genome. The RIP shows that hnRNPA1 Ab but not IgG (control) pulldown RNA sequences enriched of *KRAS* 5′UTR. *n* = 3, independent experiments, *t*-test was performed. (**D**) RIP with 293T cells stably transduced with the lentiviral vectors described in Fig. 1D. The hnRNPA1 enrichment in cells containing *KRAS*-5′UTR, *KRAS*-5′UTRmut, *KRAS*-5′UTR(D1DC); *KRAS*-5′UTR(DZDC) and *KRAS*-5′UTR(D1DZ) is shown. *n* = 3, independent experiments, Dunn’s multiple comparison test was performed. (**E**) Electrophoretic mobility shift assay (EMSA) showing binding of hnRNPA1 to 41-mer oligoribonucleotide containing the utr-c and utr-z motifs (41-mer 5′UTR), but not to the 41-mer Mut with mutated rG4 motifs. Increasing ratios of hnRNPA1:41-mer 5′UTR were used (1:10, 1:1, 10:1). (**F**) Typical ITC for the interaction of utr-c with UP1 (the proteolytic fragment of hnRNPA1 that retains rG4 binding and unfolding activity). (**G**) Best-fitting binding curves obtained for the binding of UP1 to utr-1, utr-z, and utr-c rG4s in 10 mM KPi buffer, 50 mM KCl.

Since hybridization of the lncRNAs to the 5′UTR sequence is expected to be hindered by the rG4 structures, the mechanism of mRNA downregulation should involve a protein capable to recognise and unfold the rG4 structures. We focused on hnRNPA1 for several reasons: (i) it is overexpressed in PDAC [30]; (ii) it regulates various aspects of mRNA metabolism [56]; (iii) it recognises and unwinds G4 DNA and rG4 structures [25, 30, 57, 58]; (iv) pull-down experiments with biotinylated rG4s and mass spectra analyses identified proteins, including hnRNPA1, involved in ribosome biogenesis and RNA metabolism according to functional enrichment analyses [59]. In light of this, we performed a RNA immunoprecipitation (RIP) assay to determine whether hnRNPA1 is indeed associated with the 5′UTR of *KRAS* mRNA under *in vivo* conditions. Following the scheme shown in Fig. 8A, we used an hnRNPA1-specific antibody and IgG (control) to pull down RNA-hnRNPA1 complexes in 293T cells. The results showed that the RNA bound by hnRNPA1 was enriched ninefold in the 5′UTR sequence containing the rG4 motifs compared to the control (Fig. 8B). Furthermore, no enrichment of *GAPDH* RNA was observed with either hnRNPA1 or IgG antibodies (negative control). We then performed a RIP assay with 293T cells stably transduced with *pLV-KRAS-5′UTR mScarlet* and obtained the same results as with wild-type cells (Fig. 8C). Consistent with previous data [30, 31], our results confirm that hnRNPA1 is specifically associated with the region of the 5′UTR of *KRAS* mRNA that contains the rG4 motifs. Next, we evaluated the hnRNPA1 enrichment in 293T cells stably transduced with lentiviral plasmids carrying *KRAS* 5′UTR mutant deletions that left only one of the rG4 motifs (two rG4 motifs were deleted). The RIP in this case showed that the hnRNPA1 enrichment is much lower compared to that observed with cells carrying wild-type *KRAS* 5′UTR. This indicates that the recruitment of hnRNPA1 to 5′UTR is efficient only when more than one rG4 structure is present in the same 5′UTR sequence (Fig. 8D).

To investigate the interaction between hnRNPA1 and a 41-mer fragment of the *KRAS* 5′UTR containing the rG4 motifs *utr-c* and *utr-z*, we performed an EMSA. As shown in Fig. 8E, the 41-mer RNA is efficiently bound by hnRNPA1 at a 1:1 molar ratio (lane 3), indicating strong and specific binding. As a control, a mutant version of the 41-mer oligoribonucleotide, unable to form rG4 structures, fails to bind hnRNPA1, even in the presence of a 10-fold molar excess of protein (lane 6). Additionally, hnRNPA1 binding to the wild-type 41-mer is substantially reduced in Li^+^ buffer, which destabilizes rG4 structures [60], even when the protein is present at a 10:1 ratio relative to the RNA (lane 8).

Finally, the interaction between utr-1, utr-z, and utr-c rG4s and UP1, the proteolytic fragment of hnRNPA1 that retains G4 binding and unfolding activity [61–63], was determined by ITC. A typical ITC thermogram for the interaction between utr-c and UP1 is shown in Fig. 8F. The binding curves gave the following parameters: *K*_D_= 1.14 × 10^−6^ and ΔG = −30.9 kJ/mol for utr-1, *K*_D_= 1.42 × 10^−6^, ΔG = −30.9 kJ/mol for utr-z rG4 and *K*_D_= 0.85 × 10^−6^ and ΔG = −34.7 kJ/mol for utr-c rG4 (Fig. 8G). These values are consistent with those reported for the interaction between TERRA rG4s and UP1 [62]. The negative values of ΔG suggests a moderate to strong binding and also suggests that UP1 has a specific affinity for the rG4s, potentially recognizing their unique structures.

### The protein hnRNPA1 unfolds the rG4 structures in the 5′UTR of *KRAS* mRNA

**Figure 9.**
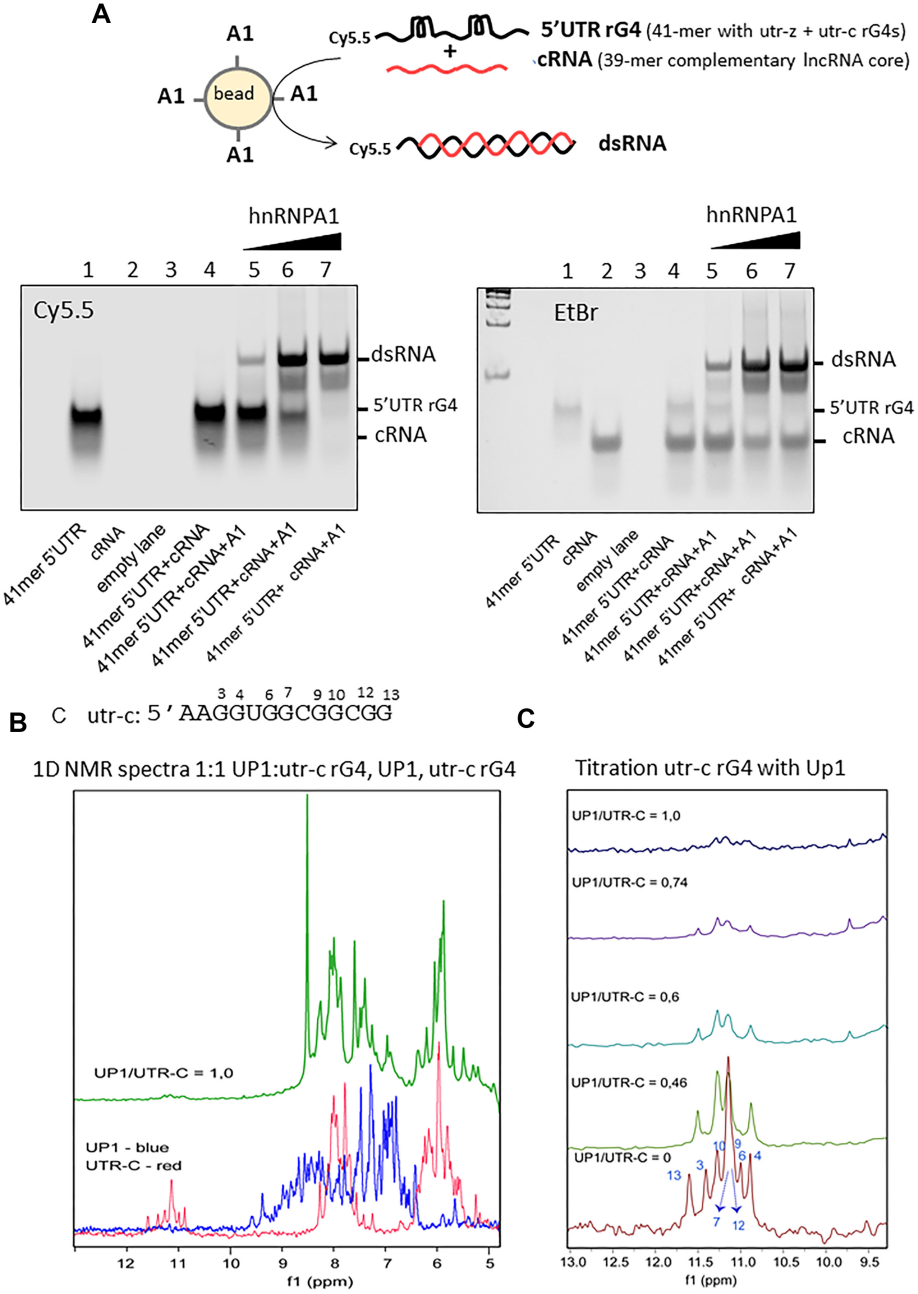
5′UTR rG4 structures are unfolded by hnRNPA1. (**A**) EMSA analysis demonstrates that hnRNPA1 unfolds and promotes hybridization between a 41-mer 5′UTR containing the utr-c and utr-z motifs and its complementary 39-mer HSALNT0012722 core sequence (referred to as cRNA). The gel on the left was visualized by the fluorescence of Cy5.5 bound to the 5′ end of the 41-mer 5′UTR. Lane 1: 41-mer 5′UTR labelled with Cy5.5 at the 5′-end; lane 2: 39-mer cRNA; lane 3: empty; lane 4: shows the 41-mer 5′UTR:cRNA mixture in a 1:2 ratio in the absence of hnRNPA1, while lanes 5, 6, and 7 show the same mixture incubated with increasing amounts of hnRNPA1 for 20 min (ratio 0.3:1, 1:1, 3:1 hnRNPA1:41mer5′UTR). hnRNPA1 promotes duplex formation in a dose-dependent manner. The same gel was then stained with 0.5 μg/ml ethidium bromide (EtBr, right panel) to visualize the migration of the cRNA strand. Electrophoresis was performed in 10 mM Tris–HCl, 50 mM KCl and 2 mM EDTA buffer at 90 V and 16°C;**B**) 1H NMR spectra of utr-c rG4 (red line), UP1 alone (blue line), and UP1:utr-c rG4 in a 1:1 ratio. The imino proton signals of utr-c rG4 between 10.8-11.5 ppm disappear in the RNA:protein complex. (**C**) ^1^H NMR titration of utr-c rG4 with increasing amounts of UP1 at UP1:utr-c rG4 ratios of 0, 0.46, 0.6, 0.74, and 1. The imino protons of guanines 3, 4, 6, 7, 9, 10, 12, and 13 of utr-c rG4 were determined (Supplementary Fig. S2). The intensity of these imino protons, which are characteristic of the rG4 structure assumed by the utr-c motif, decreases in a dose-dependent manner in the presence of increasing amounts of UP1, indicating unfolding of rG4 by the protein.

The ability of hnRNPA1 to unfold rG4 structures within the *KRAS* 5′UTR was assessed by EMSA (Fig. 9A). The 41-mer 5′UTR oligoribonucleotide with the utr-c and utr-z rG4 motifs, labelled at the 5′ end with Cy5.5, was incubated with a complementary 39-mer oligoribonucleotide corresponding to the core sequence of the lncRNA *HSALNT0012722*. The reaction was carried out for 20 min at 23°C in the presence of magnetic beads functionalized with GST-tagged hnRNPA1.

This assay is based on the principle that hnRNPA1 promotes hybridization between complementary RNA (cRNA) strands by unfolding inhibitory rG4 structures. Following incubation, the samples were analysed by polyacrylamide gel electrophoresis. Cy5.5 visualization revealed that, in the absence of hnRNPA1, the 41-mer 5′UTR remained predominantly monomeric, despite the presence of a 2:1 molar excess of cRNA (lane 4). However, upon the addition of increasing concentrations of GST-hnRNPA1, efficient hybridization between the 41-mer 5′UTR and cRNA was observed in a dose-dependent manner. Ethidium bromide staining confirmed the presence of cRNA in the gel. These results indicate that hnRNPA1 facilitates the conversion of 5′UTR with its rG4 structure into a duplex form, consistent with effective unfolding of the rG4 elements by hnRNPA1.

To further validate this mechanism, the ability of UP1 to unfold rG4s was examined by NMR spectroscopy. One-dimensional ^1^H NMR spectra were acquired for UP1 alone, utr-c rG4 alone, and a 1:1 utr-c: rG4:UP1 complex (Fig. 9B). In the presence of UP1, the imino proton peaks characteristic of the folded utr-c rG4 structure disappear, indicating complete unfolding upon complex formation. The rG4-specific imino signals, located around 11 ppm, do not overlap with protein resonances, ruling out spectral interference and confirming genuine disruption of the rG4 structure. Eight distinct imino peaks representing the rG4 were annotated in the utr-c spectrum (see Supplementary Fig. S2).

To investigate the unfolding process in more detail, a titration of UP1 with utr-c rG4 was performed at molar ratios of UP1:utr-c = 0, 0.46, 0.60, 0.74, and 1.0 (Fig. 9C). At zero protein concentration, the rG4 exhibited five well-resolved imino peaks (corresponding to nucleotides 13, 3, 10, 6, and 4) and three overlapping signals (nucleotides 7, 9, and 12) (Supplementary Fig. S2). As the UP1 concentration increased, the intensity of these peaks diminished progressively, disappearing entirely at a 1:1 ratio: a further evidence that UP1 induces unfolding of the rG4 structure. Comparable NMR results were obtained for the utr-z rG4 motif (Supplementary Fig. S10), supporting the conclusion that UP1 efficiently unfolds both rG4 elements.

Taken together, the EMSA and NMR data support a mechanistic model in which hnRNPA1 initially binds to and unwinds rG4 structures within the *KRAS* 5′UTR, thereby facilitating hybridization with the complementary lncRNA to form an RNA duplex. Although hnRNPA1 exhibits little to no affinity for linear RNA, our results suggest that the protein remains associated with the 5′UTR during duplex formation. This implies that the rG4 region bound by hnRNPA1 may not be fully unfolded, despite the disappearance of imino proton signals in the NMR spectra. These observations suggest that hnRNPA1 binding is initially dependent on the rG4 scaffold, with dissociation likely occurring only after successful duplex formation. Thus, rG4-mediated recruitment of hnRNPA1 represents a critical step in 5′UTR remodelling and RNA–RNA hybridization.

### hnRNPA1 facilitates base pairing between 5′UTR *KRAS* and lncRNA HSALNT0012722 *in vivo*

**Figure 10.**
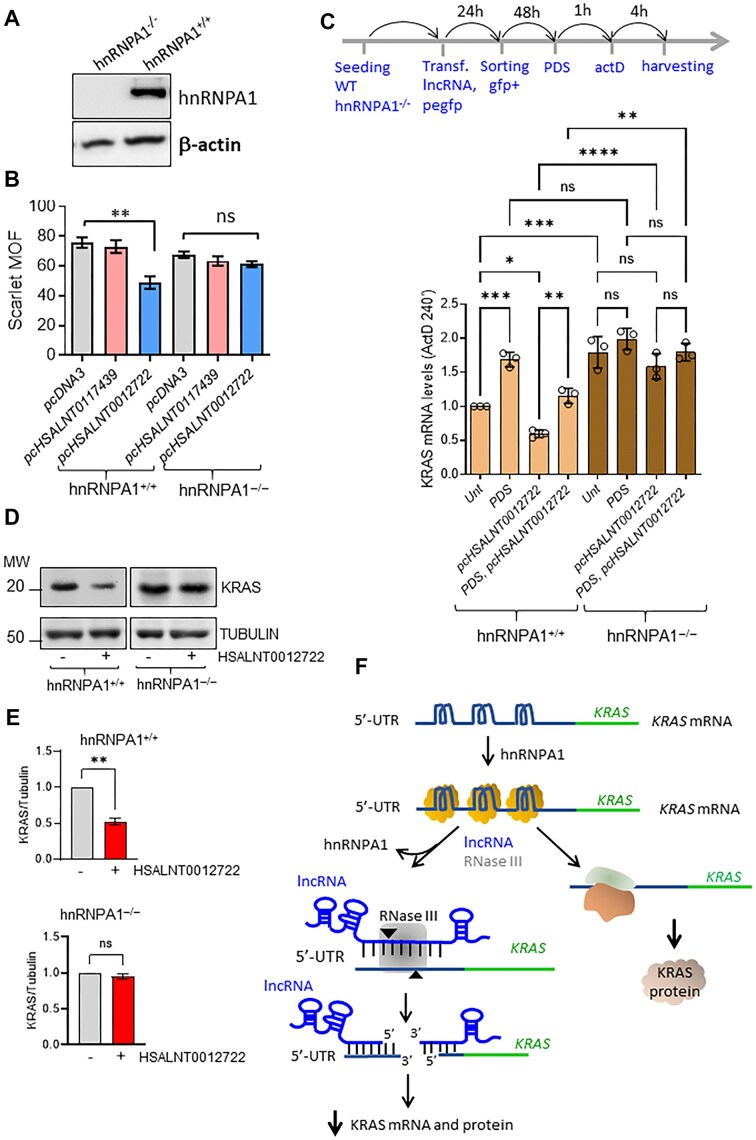
Effect of hnRNPA1 on Panc-1 cells overexpressing the lncRNA HSALNT001272 and mechanism controlling *KRAS* transcription and mRNA stability. (**A**) Western blot showing hnRNPA1 expression in the indicated clones of Panc-1 cells; the *hnRNPA1*^*−/−*^ clone does not produce hnRNPA1. (**B**) The *pLV-KRAS-5′UTR mScarlet* reporter was integrated into *hnRNPA1*^*+/+*^ and *hnRNPA1*^*−/−*^ Panc-1 cells. Founder cells were co-transfected with 2 μg *pcDNA3* plasmids expressing HSALNT0117439 or HSALNT0012722 and 500 ng pEGFP C1 with 6 μl Lipofectamine 2000. After 48 h, the GFP + cells were scored for mScarlet positivity by flow cytometry. Data are expressed as MOF in the fraction of GFP + cells. *n* = 3, independent experiments, *t*-test was performed. (**C**) *KRAS* mRNA levels in Panc-1 cells overexpressing or not HSALNT001272. Cells were processed as described in the experimental scheme and harvested 4 h after treatment with ActD. For simplicity wild-type (*hnRNPA1*^*+/+*^) Panc-1 cells are called A1^+/+^, whereas Panc-1 cells in which hnRNPA1 was knockout (*hnRNPA1*^*−/−*^) are called A1^−/−^. Data are expressed as mean ± SD, normalised to wild-type Panc-1 cells (A1^+/+^) treated with ActD only (indicated as “Untreated”, Unt). *n* = 3, independent experiments. Dunn’s multiple comparison test. (**D**) Western blot showing KRAS protein levels in WT and hnRNPA1^−/−^ cells in which the lncRNA was overexpressed, as indicated. Tubulin was used as loading control. (**E**) Densitometric quantification of three biological replicates of immunoblot experiments described in Fig. 10D. Data are expressed as KRAS/Tubulin relative levels. Mean ± SD, *n* = 3, independent experiments. (**F**) Proposed mechanism for fine-tuning *KRAS* mRNA levels in Panc-1 cells. The mechanism involves rG4 structures in the 5′UTR, lnc RNA complementary to the 5′UTR sequence of mRNA containing the rG4 motifs and protein hnRNPA1 as rG4 unfolding protein.

Given the capacity of hnRNPA1 to unfold the 5′UTR rG4 structures of *KRAS*, we asked if the protein facilitates the base pairing between the lncRNA HSALNT0012722 and the 5′UTR mRNA under *in vivo* conditions. For this purpose, we used a CRISPR/Cas9-engineered Panc-1 clone lacking hnRNPA1 expression (*hnRNPA1*^*−/−*^), which was previously produced in our laboratory [30] (Fig. 10A). The effect of HSALNT0012722 on mScarlet protein levels was measured in *hnRNPA1*^*−/−*^ and wild-type *hnRNPA1*^*+/+*^ Panc-1 cells stably transduced with *pLV-KRAS-5′UTR mScarlet*. The results showed that HSALNT0012722 reduced mScarlet protein (by 36%, P < 0.01) in wild-type *hnRNPA1*^*+/+*^ cells but not in *hnRNPA1*^*−/−*^ cells. In the latter, HSALNT0012722 had no effect, consistent with the fact that it cannot hybridise to the 5′UTR because the rG4 structures cannot be unfolded in *hnRNPA1^−/−^*cells (Fig. 10B).

Next, we examined the ability of HSALNT0012722 to decrease *KRAS* mRNA levels in *hnRNPA1*^*+/+*^ and *hnRNPA1*^*−/−*^ Panc-1 cells after transcriptional blockade with ActD (see Fig. 10C). In *hnRNPA1^+/+^*cells, treatment with PDS led to a significant increase in *KRAS* mRNA levels from 1.0 to 1.7 (*P* < 0.001), which is likely due to PDS-mediated stabilization of 5′UTR rG4 structures, blocking their unwinding by hnRNPA1 and preventing hybridization with HSALNT0012722. Conversely, transfection of *hnRNPA1*^*+/+*^ cells with pcHSALNT0012722 resulted in ∼50% reduction in *KRAS* mRNA levels (*P* < 0.05), consistent with lncRNA hybridization to the 5′UTR and promotion of mRNA degradation. This reduction was abolished by co-administration of PDS, presumably because PDS inhibits the formation of the RNA duplex required for degradation. Under this condition, *KRAS* mRNA level should theoretically be consistent with that observed with PDS alone; however, it remained somewhat lower, suggesting that some lncRNA can still access the target 5′UTR in the presence of PDS. *hnRNPA1*^*−/−*^ cells had a significantly higher basal level of *KRAS* mRNA than *hnRNPA1*^*+/+*^ cells (1.8 versus 1.0; *P* < 0.01), consistent with the absence of hnRNPA1-mediated unwinding of rG4s, thereby hindering lncRNA-induced degradation. As expected, neither PDS nor HSALNNT0012722 had any effect on *KRAS* mRNA levels in *hnRNPA1^−/−^*cells, supporting a model in which rG4s remain locked and inaccessible in the absence of hnRNPA1.

Finally, we examined KRAS protein levels in both cell types with and without pcHSALNT0012722 transfection (Fig. 10D and E). Expression of lncRNA reduced KRAS protein levels by ∼50% in *hnRNPA1*^*+/+*^ cells but, as expected, had no effect in *hnRNPA1*^*−/−*^ cells. Taken together, these results support a model in which hnRNPA1 and HSALNT0012722 jointly regulate *KRAS*mRNA stability by remodelling the 5′UTR-rG4 structures, as shown in Fig. 10F.

### Transcriptional and post-transcriptional regulation of oncogenic *KRAS*

This study shows that oncogenic *KRAS* is regulated at both transcriptional and posttranscriptional levels. Previously, we have shown that PARP-1 binds to G4 structures in the *KRAS* promoter, specifically the 32R and G4-mid structures, with the 32R structure being favoured [24, 25]. After binding to these G4 structures, PARP-1 undergoes auto-PARylation, giving it an anionic charge [29]. We proposed that the G4:PARP-1 complex could serve as a recruitment platform for TFs such as hnRNPA1 and MAZ, which are cationic under physiological conditions (pI > 7.4). The cellular levels of *KRAS* mRNA are tightly regulated to maintain homeostasis and ensure proper function. While G4 DNA structures are central to transcriptional activation, additional elements such as 5′UTR rG4s, lncRNAs and hnRNPA1 play a crucial role in the post-transcriptional regulation of *KRAS*. Recent findings emphasise the multiple functions of lncRNAs in translational regulation. These include the function as decoys for translation factors [64], sponging tumor- suppressor miRNAs [65] or the direct interaction with target mRNAs to form double-stranded helices [66]. For example, the lncRNA MALAT1 promotes cancer development and proliferation by acting as a molecular sponge for miR-217, a known inhibitor of *KRAS* [67]. This enhances *KRAS* signalling and drives PDAC oncogenesis [68]. Similarly, LINC00460 acts as a competitive endogenous RNA by sequestering miRNAs targeting *KRAS* mRNA, thereby preventing translational repression. LINC00460 has been associated with tumourigenesis and cancer progression, particularly in colorectal and lung cancer [69].

In this study, we identified novel lncRNAs transcribed from the LINC01750 locus, including HSALNT0012722, HSALNT0012723 ad LINC01750:9, which pair with the 5′UTR of *KRAS* mRNA to promote its degradation. The lncRNA appears to function as a *KRAS*-specific tumour suppressor. The 5′UTR rG4 structures inherently increase RNA stability by protecting the RNA. However, our results—which are consistent with a recent study by Lee *et al.* [70]—suggest a more complex regulatory mechanism in pancreatic cancer cells involving rG4s, lncRNA, and hnRNPA1. The protein hnRNPA1, known for its G4 unfolding activity, facilitates the hybridization between the 5′UTR of *KRAS* mRNA with the lncRNAs from the LIN017250 locus, which form a double-stranded RNAs that are recognised and degraded by the dsRNA-specific endoribonuclease RNase III. This mechanism is supported not only by the transfection of cells with a vector producing the core of lncRNAs, but also by the stimulation or suppression of the transcription of the endogenous lncRNAs from the LINC01750 locus by the dCas9/VP64 and dCas9/KRAB system, respectively.

We also investigated whether lncRNAs transcribed from the LINC01750 locus might potentially target genes other than *KRAS*. To explore this, we performed a BLAST analysis (blastn suite, RRID:SCR_001598) using the reverse complement of the core sequence of HSALNT0012722, which is complementary to the 5′UTR of *KRAS* mRNA, against the entire human transcriptome. This in silico analysis identified seven potential targets with E-values below the significance threshold (<1e-5). Among these, *SRP68* exhibited the highest alignment score and expression level after *KRAS* (Supplementary Fig. S11A). However, the region of *SRP68* complementary to the lncRNA contains four mismatches within a sequence analogous to the utr-z region of *KRAS* 5′UTR where binding is predicted to occur. Functional assays revealed that *SRP68* expression showed only a minimal response to lncRNA activation via dCas9/VP64, and no response to repression by dCas9/KRAB, suggesting it is unlikely to be a real off-target (Supplementary Fig. S11B). The remaining identified transcripts displayed lower alignment scores than *SRP68*, further reducing the likelihood that they are functionally targeted by LINC01750 lncRNAs.

In conclusion, the regulatory mechanism of *KRAS* described in this study ensures precise control of mRNA levels, thereby maintaining balanced protein production and preventing aberrant gene expression. Dysregulation of lncRNA-mediated mRNA degradation has been found in a number of diseases, including cancer, neurodegenerative disorders, and immune disorders [71]. Further research is required to elucidate the precise molecular steps underlying this complex regulatory network. A deeper understanding of the pathways governing *KRAS* mRNA stability, transcription, and translation will be essential for developing targeted therapies for diseases driven by aberrant *KRAS* signaling.

## Supplementary Material

gkaf886_Supplemental_File

## Data Availability

The data underlying this article are available in the article and in its online supplementary material. Raw data of RIP-seq experiments have been uploaded with GEO, accession GSE283011. H3K27ac Chip-seq data are accessible through GEO (GSE235472). A UCSC browser session can be accessed at https://genome.ucsc.edu/s/eros.digiorgio/rG4.

## Supplementary data

Supplementary data is available at NAR online.
