# Supplementary material for: Post-transcriptional control of KRAS: functional roles of 5′UTR RNA G-quadruplexes, long noncoding RNA, and hnRNPA1
Source: Nucleic Acids Res. 2025 Sep 18;53(17):gkaf886. doi: 10.1093/nar/gkaf886 (PMC12449053; doi:10.1093/nar/gkaf886)
Supplement: gkaf886_Supplemental_File [file gkaf886_supplemental_file.pdf]

Supplementary Data

Post-transcriptional control of KRAS: Functional roles of 5’UTRRNA G-quadruplexes, long non-coding RNA and hnRNPA1

Ylenia Cortolezzis[1], Zahraa Othman[2,&], Francesca Agostini[1], Iman Ibrahim[2] Raffaella Picco[1]; Gilmar F. Salgado[2,*], Eros Di Giorgio[1,*] and Luigi E. Xodo[1,*]

Supplementary Table S1. List of the primers used for qRT-PCR experiments and RNA oligonucleotides used in this study.

Oligo name	Sequence (5'->3')	Application
**HPRT_qPCR_qPCR_FW**	AGACTTTGCTTTCCTTGGTCAGG	qPCR
**HPRT_QPCR_qPCR_RV**	GTCTGGCTTATATCCAACACTTCG	qPCR
**GAPDH_qPCR_FW**	CCCTTCATTGACCTCAACTACATG	qPCR
**GAPDH_qPCR_RV**	TGGGATTTCCATTGATGACAAGC	qPCR
**luc_qPCR_FW**	GGTCCTATGATTATGTCCGGTTATGT	qPCR
**luc_qPCR_REV**	CGTTCTTCGTCCCAGTAAGCTATGT	qPCR
**mSCARLET_qPCR_FW**	AAGCTGAAGGTGACCAAGGG	qPCR
**mSCARLET_qPCR_RV**	GCCGTCCTCGAAGTTCATCA	qPCR
**HSALNT0012722_qPCR_FW**	GAATCCTGGATCTCGGCCTG	qPCR
**HSALNT0012722_qPCR_RV**	CTCTCCACACCCAGAAA	qPCR
**KRAS_CHIP_FW**	TCTTCGCAGCTTCTCTGTGG	CHIP
**KRAS_CHIP_RV**	CGCCAATTCTGACCCGGAG	CHIP
**GAPDH_CHIP_FW**	GCTTGCCCTGTCCAGTTAAT	CHIP
**GAPDH_CHIP_RV**	TAGCTCAGCTGCACCCTTTA	CHIP
**Utr1**	GGCGGCGGAGG	synthetic RNA
**UtrC**	GGUGGCGGCGG	synthetic RNA
**Utrz**	GGCGGCGGCAGUGGCGGCGG	synthetic RNA
**HSALNT0117439 (42-mer)**	CUGCUGCUGCUGCUGCUGCUGCCG-CCGCCGCCGCCGGUUCUU	synthetic RNA
**HSALNT0012722 (39-mer)**	GCCGCCGCCACUGCCGCCGCCACUGCCGCCGCCGCCGCU	synthetic RNA
**41-mer 5’UTR-Cy5.5**	GCAGCGGCGGCGGCAGUGGCGGCGGC-GAAGGUGGCGGCGGC	synthetic RNA
**41-mer 5’UTRmut**	GCAGCGCCAGCCGCAGUCGCGUCCGCG-AACGUAGCCGCCGC	synthetic RNA

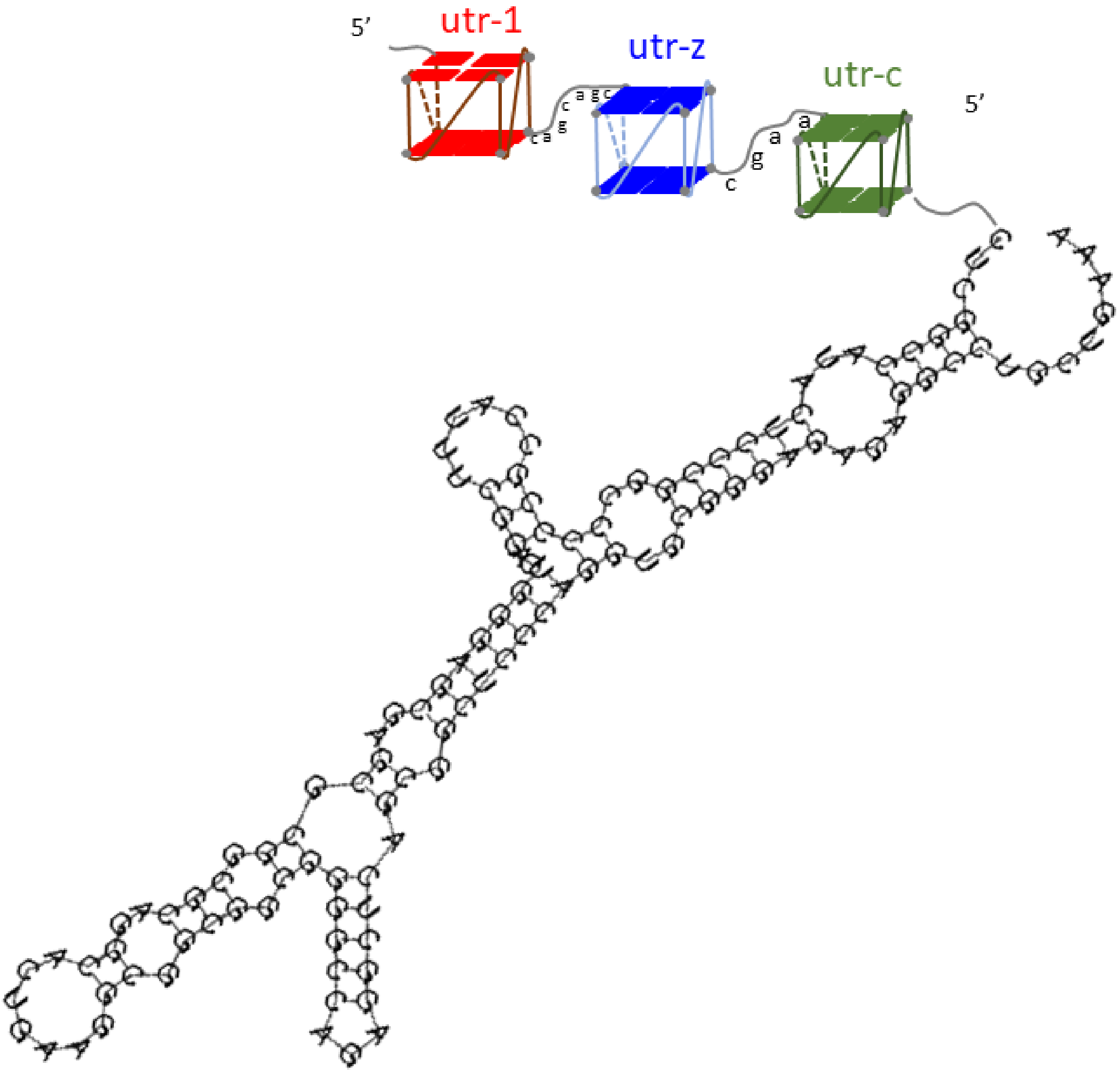

Supplementary Fig. S1. Sequence of 5′UTR of *KRAS* mRNA. The three rG4 motifs with G-score=21 identified by QGRS Mapper are indicated in colours: red (utr-1), blue (utr-z) and green (utr-c). The 3 rG4 motifs lie within the first 80 nt of the 5′UTR. The remaining part of 5′UTR assumes a hairpin loop structure according to the RNAfold software 2.7.0.

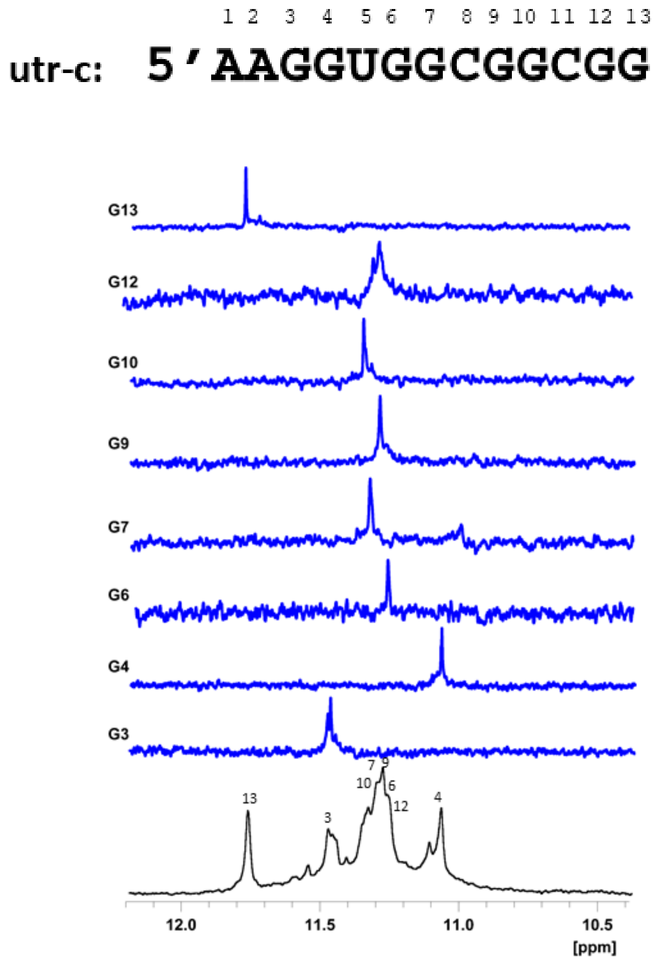

Supplementary Fig. S2: Imino proton fingerprint spectrum of the rG4 motif utr-c, which adopts a stable rG4 structure supported by two stacked G-tetrads.

5’UTR:CTAGGCGGCGGCCG**CTGCCGCTGACG**CAGCAGC**GCCAGCCGCAGTCGCGTCCG**CGA**AC**
GTAGCCGCCGCTCGGCCAGTACTCCCGGCCCCCGCCATTTCGGACTGGGAGCGAGCGCGGCGCA
GGCACTGAAGGCGGCGGCGGGGCCAGAGGCTCAGCGGCTCCCAGGTGCGGGAGAGAGGCCTGCT
GAAA3’

Supplementary Fig. S3: Sequence of the KRAS 5’UTRmut with the three mutated rG4 motifs (utr1, red), utr-z (blue) and utr-c (green). The point mutations are highlighted in yellow.

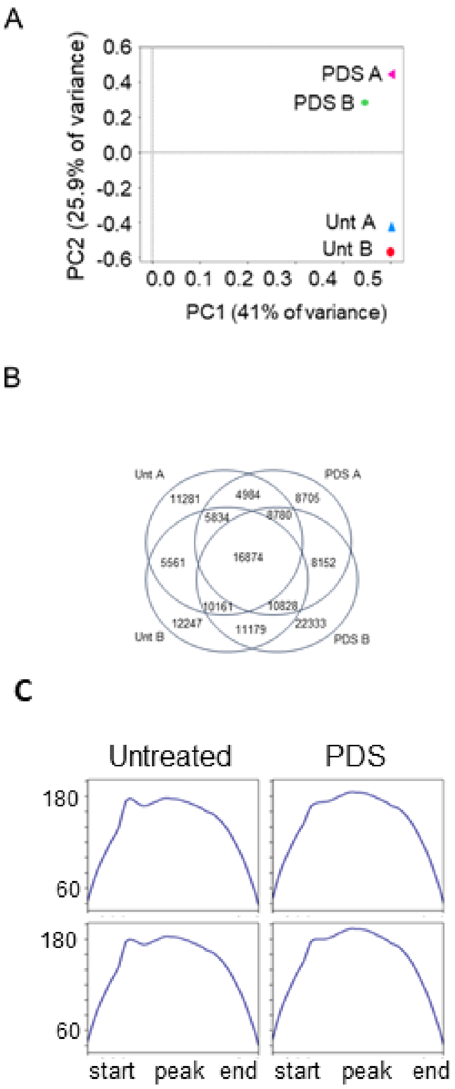

Supplementary Fig. S4: (A) Bidimensional Principal Component (PCA) analysis generated from indicated bigwig rG4 data. The RNA samples were reverse transcribed into cDNA and analysed by next-generation sequencing. Biological replicates showed high concordance under both untreated and PDS-treated conditions; (B) A Venn diagram illustrating the number of significantly enriched peaks (defined as enrichment > 2 and FDR < 0.05) that are shared between untreated and PDS-treated samples, as well as those unique to each condition; (C) Profile of rG4 signal 1 kb around the 16874 peaks enriched in both untreated and PDS-treated Panc-1. Replicate A above, replicate B below.

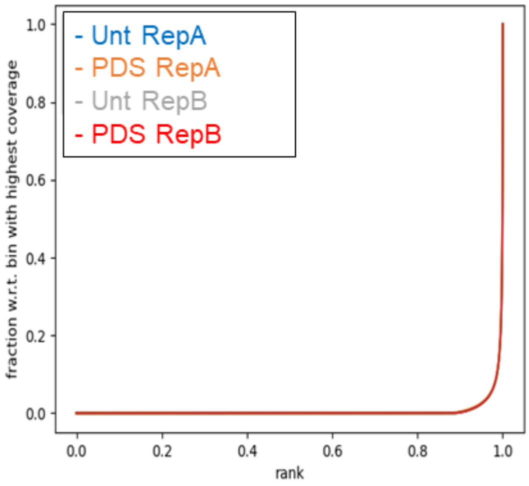

Supplementary Fig. S5. A fingerprint plot was generated using the specified .bam files for the indicated samples to assess the distribution of sequencing reads across the genome, providing insights into data quality and potential biases in chromatin accessibility or enrichment patterns.

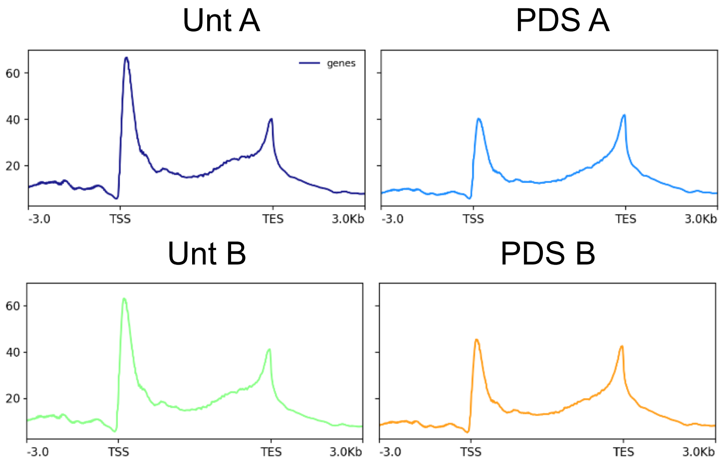

Supplementary Fig. S6. Distribution analysis relative to transcription start sites (TSS) and transcription end sites (TES) revealed a bimodal enrichment of rG4 structures at the 5′UTR and 3'UTR.

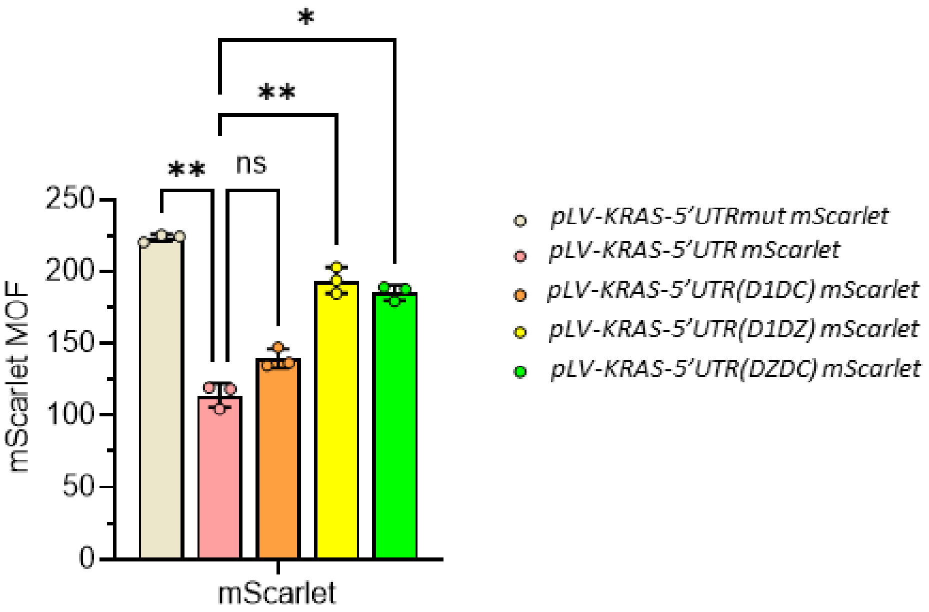

Supplementary Fig. S7. mScarlet mean optical fluorescence (MOF) in 293 cells expressing mScarlet under the control of the 5'utr indicated in the legend and described in Figure 4D. Data are expressed as mean ± SD, n=3, independent experiments. Dunn's multiple comparison test was performed

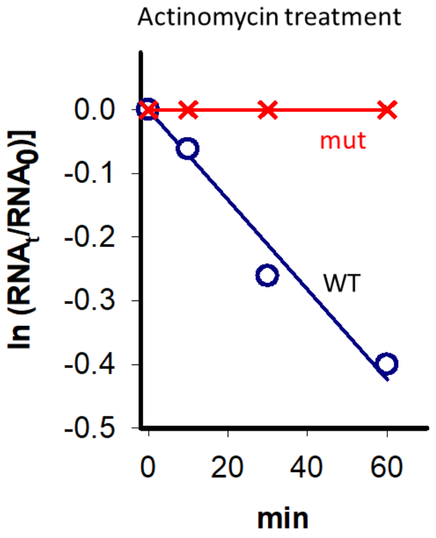

Supplementary Fig. S8. 293T WT or mut (lacking the 5'utr) cells were treated with 20 nM ActD as described in Fig. 4A and harvested at the indicated time points after treatment. The plot shows that ln[RNA]t/[RNA]0 versus time after ActD treatment is a straight line, indicating that the decay of mRNA in the 293T wild-type cells follows first-order kinetics.

The CRISPR/Cas9-edited 293T cells lacking the utr-c and utr-z motifs exhibit stable mRNA levels over the 60-minute period.

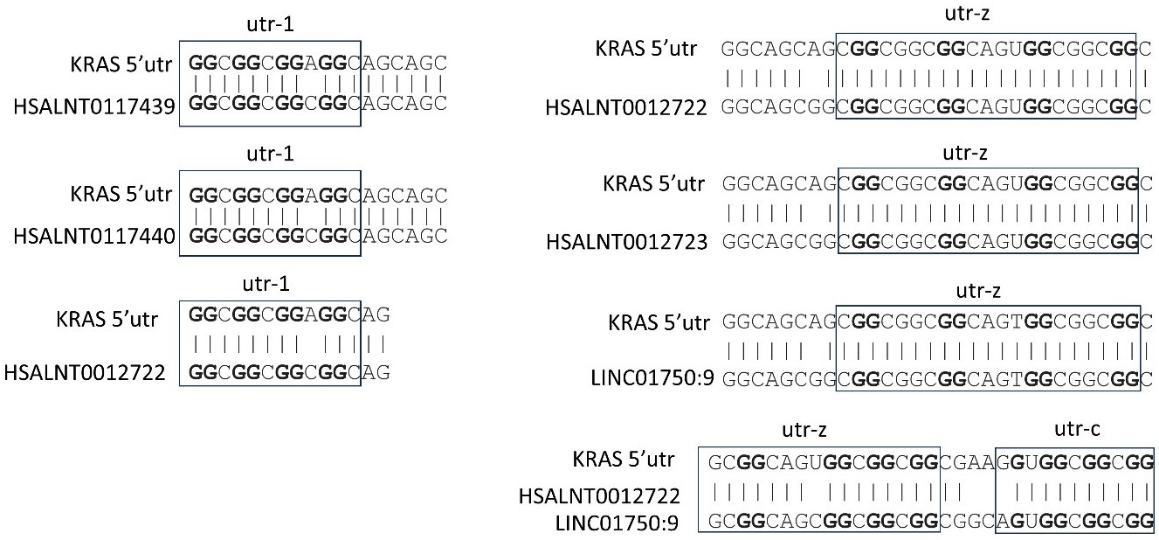

Supplementary Fig. S9. (Right) Alignment of the reverse complementary sequence of the lncRNAs transcribed from the LINC01750 locus (HSALNT0012722, HSALNT0012723 and LINC01750:9) to the KRAS 5′utr-z (and utr-c) sequence; (Left) Alignment of the lncRNAs transcribed from the LINC01750 locus (HSALNT0117439, HSALNT0117440 and HSALNT0012722) to the KRAS 5′utr-1.

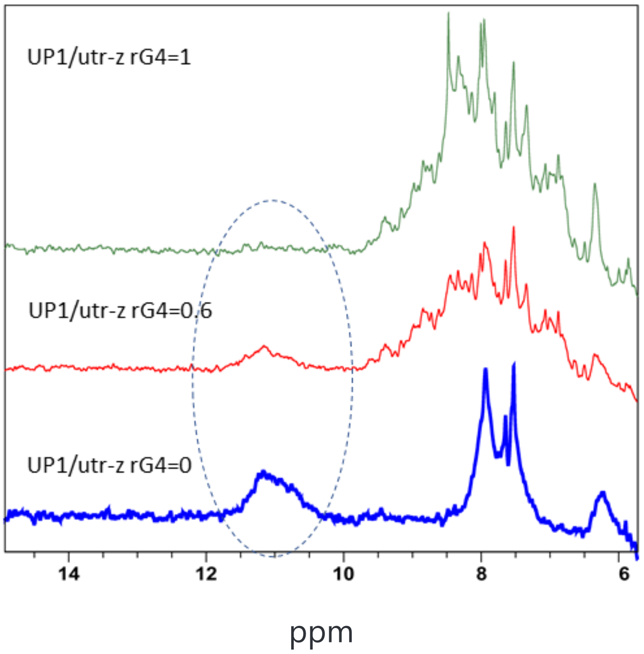

Supplementary Fig. S10: 1H-NMR iminoprotons of utr-z rG4 alone and mixed with UP1 at UP1/rG4 ratios of 0.6 and 1. In the presence of UP1, the intensity of the iminoprotons is reduced in a dose-dependent manner. In the 1:1 UP1/rG4 complex, the iminoproton signals disappeared completely.

(A)

Table A: Potential targets of lncRNAs from the LINC01750 locus identified within the entire human transcriptome

ID	Gene name	E-value	Transcripts RPKM	Max score
NM_033360.4	KRAS	2e-6	48.84	42.1
NM_001260502.2	SRP68	3e-6	114.6	40.1
NM_005859.5	PURA	5e-6	12.05	39.1
NM_006226.4	MCCL1	2e-5	3.04	38.2
NM_001163474.2	ZNF746	2e-5	17.96	38.2
NM_001426.4	EN1	2e-5	0.18	38.2
NM_002505.5	NFYA	2e-5	33.37	38.2

(B)

```
KRAS 5'utr     GGCAGCAGCGGCGGCGGCAGUGGCGGCGGC
               ||||||X|||||||||||||||||||||||
HSALNT0012722  GGCAGCGGCGGCGGCGGCAGUGGCGGCGGC

SRP68          GCGGGCGGCGGCGGCGGCAGUGGCGGCGGC
               |XXX||||||||||||||||||||||||||
HSALNT0012722  GGCAGCGGCGGCGGCGGCAGUGGCGGCGGC
```

(C)

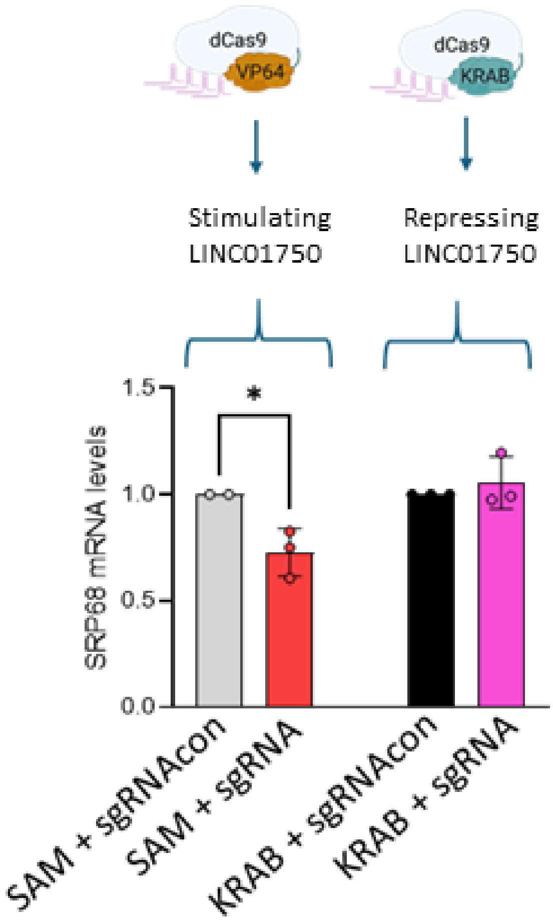

Supplementary S11. (A) Results of the BLAST analysis (blastn suite, RRID:SCR_001598) using the reverse complement of the core sequence of HSALNT0012722, which is complementary to the 5′UTR of *KRAS* mRNA, against the entire human transcriptome; (B) Alignment of the reverse complement of the core sequence of HSALNT0012722 at the 5′UTR of KRAS mRNA and SRP68 mRNA); (C) sRP68 mRNA levels in 293T cells treated with either the dCas9/VP64 system, which stimulates expression of lncRNAs of the LINC01750 locus, or with the dCas9/KRAB system, which instead represses LINC01720 expression. While the dCas9/VP64 system downregulates SRP68 mRNA levels in 293T cells by ~25%, *KRAS* mRNA is downregulated by ~50%. Instead, dCas9/KRAB has no effect on SRP68 mRNA, while it upregulates *KRAS* mRNA by ~25 % in 293T cells and by ~70 % in PDAC cells (MIAPaCa2). The putative off-target gene that had a match score closest to that of *KRAS* showed a minimal response to dCas9/VP64 and no response to dCas9/KRAB, suggesting that it is unlikely to be a true off-target gene. Data are expressed as mean ± SD, n=3, independent experiments. A *t* test was performed
